# PGpath: recommending population-adapted linear reference genomes from pangenome subpaths

**DOI:** 10.1093/bib/bbag547

**Published:** 2026-10-11

**Authors:** Zhengfa Xue, Xuwen Wang, Jiajing Yuan, Tianci Wang, Xiaoqi Sheng, Tinghe Zhang, Jiayin Wang, Hongmin Cai

**Affiliations:** School of Future Technology, South China University of Technology, No. 777 Xingye Avenue East, Panyu District, Guangzhou, Guangdong 511442, China; The Comprehensive Breast Care Center, The Second Affiliated Hospital of Xi’an Jiaotong University, No. 157 Xiwu Road, Xincheng District, Xi’an, Shaanxi 710004, China; School of Computer Science and Technology, Faculty of Electronics and Information Engineering, Xi’an Jiaotong University, No. 28 Xianning West Road, Beilin District, Xi’an, Shaanxi 710049, China; Shaanxi Engineering Research Center of Medical and Health Big Data, Xi’an Jiaotong University, No. 28 Xianning West Road, Beilin District, Xi’an, Shaanxi 710049, China; The Comprehensive Breast Care Center, The Second Affiliated Hospital of Xi’an Jiaotong University, No. 157 Xiwu Road, Xincheng District, Xi’an, Shaanxi 710004, China; School of Future Technology, South China University of Technology, No. 777 Xingye Avenue East, Panyu District, Guangzhou, Guangdong 511442, China; School of Future Technology, South China University of Technology, No. 777 Xingye Avenue East, Panyu District, Guangzhou, Guangdong 511442, China; School of Computer Science and Technology, Faculty of Electronics and Information Engineering, Xi’an Jiaotong University, No. 28 Xianning West Road, Beilin District, Xi’an, Shaanxi 710049, China; Shaanxi Engineering Research Center of Medical and Health Big Data, Xi’an Jiaotong University, No. 28 Xianning West Road, Beilin District, Xi’an, Shaanxi 710049, China; School of Future Technology, South China University of Technology, No. 777 Xingye Avenue East, Panyu District, Guangzhou, Guangdong 511442, China

**Keywords:** reference genome, pangenome subpath, K-mer features, large-scale population sequencing, genetic diversity

## Abstract

Large-scale population sequencing requires reference genomes that accurately represent the genetic background of target populations. Although high-quality linear references such as T2T-CHM13 have improved the completeness of human genome representation, a single reference genome remains insufficient to capture genetic diversity across populations. Pangenome graphs provide a richer representation of multi-source genomic variation, but translating pangenome diversity into practical reference genomes remains challenging. First, the direct application of pangenome graphs in routine large-scale sequencing analysis is limited by graph complexity and poor compatibility with existing linear-reference workflows. Second, existing pangenome-driven personalized references mainly target individual samples rather than populations, and current path recommendation strategies may overlook local source consistency during branch-node selection. In this study, we propose PGpath, a pangenome-based method for recommending population-adapted linear reference genomes. PGpath generates a reference from a selected pangenome subpath, preserving pangenome diversity while retaining a linear format compatible with standard downstream pipelines. In this study, we characterize the shared genetic background of samples using population-level k-mer frequency features and reformulate reference recommendation as branch-node prediction in the pangenome graph. To encourage local source consistency, we develop a topology-constrained multi-task neural network incorporating source-consistency relationships between adjacent branch nodes. Experiments on Human Pangenome Reference Consortium (HPRC) population datasets and HG002 variant detection benchmarks showed that PGpath-derived references improved read mapping performance and reduced downstream variant detection errors compared with commonly used references. PGpath provides a practical strategy for translating pangenome diversity into population-adapted linear references for large-scale genomic analysis.

## Introduction

Large-scale population sequencing is increasingly used in clinical screening and cohort-based genomic studies, including non-invasive prenatal testing [1], tumor screening [2], disease cohort analysis, and population genetic research [3]. In these applications, large numbers of sequencing samples are typically processed through unified workflows for read mapping, variant detection, and comparative analysis. The accuracy of these workflows depends strongly on the choice of reference genome [4]. When the reference genome does not adequately represent the genetic background of the target population, systematic genetic differences between the reference and the target population can lead to read mapping bias, false-positive and false-negative variant calls, and distorted allele frequency estimates [5]. Therefore, constructing reference genomes that better match target populations is important for improving the reliability of large-scale genomic analysis.

Over the past two decades, human reference genomes have progressively evolved from GRCh37 and GRCh38 to the more complete Telomere-to-Telomere (T2T) assembly series, establishing a stable coordinate framework and a unified analytical baseline for genomic research (Fig. 1A) [6]. However, a single linear reference genome still represents only a limited genetic background and cannot fully capture sequence diversity across populations [7]. A pangenome provides a richer reference representation by integrating multiple genomes or haplotypes into a graph structure composed of nodes, edges, and paths [8, 9]. Such representations can incorporate allelic variants, insertions and deletions, and structural variants from diverse individuals and populations. When a pangenome graph contains haplotypes or variant sequences that are closer to the target sample, graph-based references can reduce alignment bias in non-reference allelic regions and improve downstream variant calling and genotyping [10, 11].

**Figure 1:**
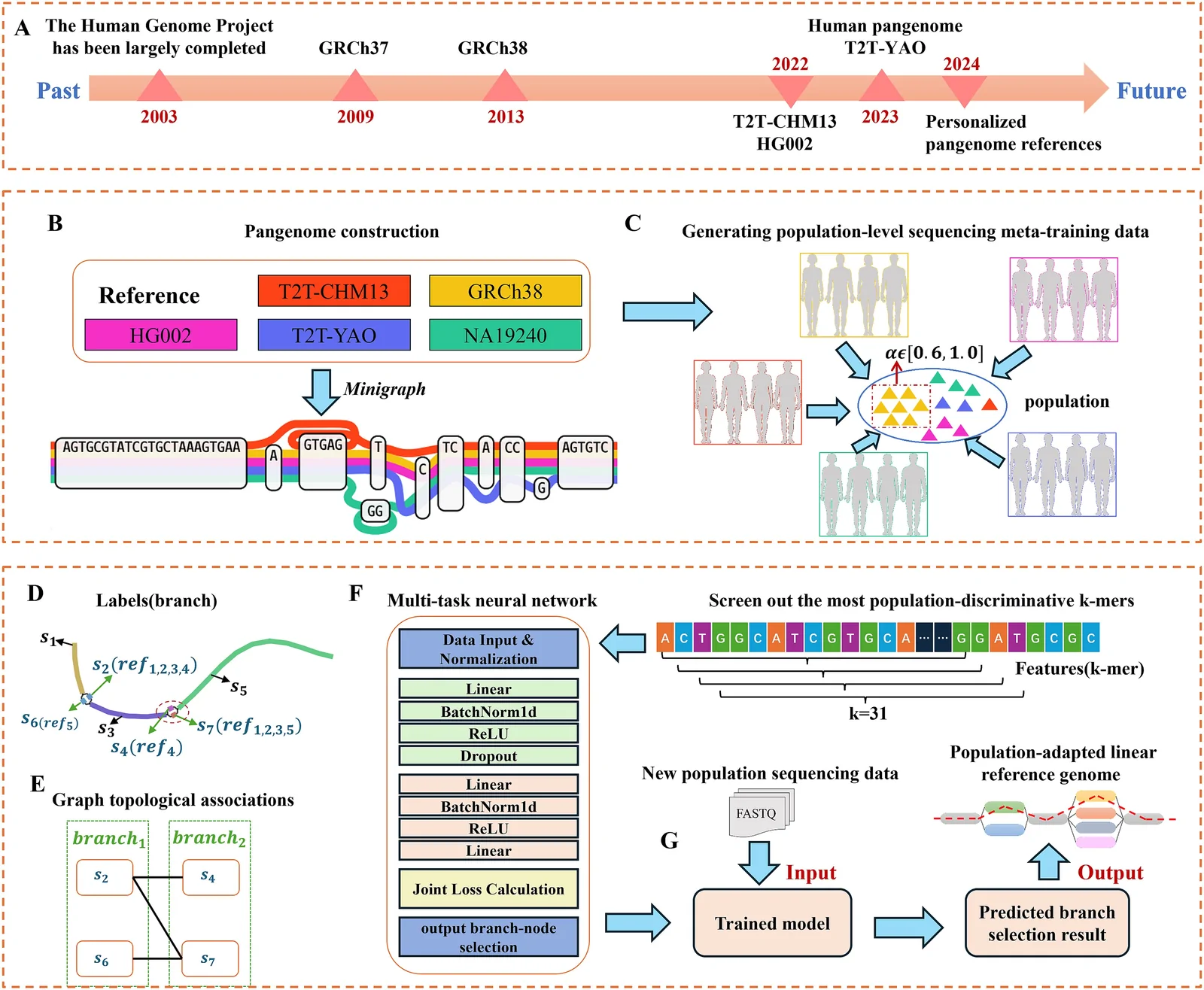
Overview of the PGpath framework. (A) Timeline of human reference genome development, from the completion of the Human Genome Project and linear references to T2T-level assemblies, human pangenome resources, and personalized pangenome references. (B) Construction of the pangenome graph from high-quality and widely used reference assemblies, including T2T-CHM13, GRCh38, HG002, T2T-YAO, and NA19240. (C) Generation of population-level sequencing meta-training data. Simulated sequencing samples from different reference sources were sampled and combined to form population-level training units with different source compositions. (D) Definition of branch labels. Each branch represents a local path decision point in the pangenome graph, and each branch node represents a candidate alternative sequence segment. (E) Construction of branch-node topological associations. Source-consistency relationships between branch nodes from adjacent branches were encoded as topological associations. (F) Topology-constrained multi-task neural network for branch-node selection. (G) Reference recommendation for a target population. Sequencing data from a new population are processed into standardized k-mer features and fed into the trained model. The predicted branch-node selections are integrated according to graph connectivity to generate a population-adapted linear reference sequence.

The direct application of pangenome graphs in routine large-scale sequencing analysis remains challenging. Compared with linear references, graph-based references represent multiple alternative alleles, indels, and structural variants as branching paths, which increases structural complexity and expands the search space for read alignment [12, 13]. As graph complexity increases, variants represented in the graph but absent from the target sample may introduce additional local paths, increase mapping ambiguity, and reduce alignment accuracy [14]. To mitigate this issue, existing strategies such as allele-frequency-based graph pruning and haplotype-aware graph alignment reduce graph complexity by filtering variants or restricting the search space to representative haplotype paths [15–17]. However, such simplification may remove true variants carried by the target population, thereby weakening the ability of the graph to represent population-adapted sequences. In addition, most genome annotations, clinical databases, variant resources, primer and probe design workflows, and standard file formats are still organized around linear reference coordinates [18]. Although tools such as *VG* [19], *minigraph* [20], *PGGB* [10], and *ODGI* [21] have advanced graph construction, alignment, and visualization, graph-based workflows remain less standardized and less easily deployable than conventional linear-reference pipelines. Therefore, graph-based reference analysis cannot yet fully replace conventional linear-reference workflows in large-scale population cohorts.

To exploit the sequence diversity in pangenome graphs while retaining the practical advantages of linear-reference workflows, a more targeted strategy is to infer a sample-adapted subgraph from the pangenome rather than directly using the complete graph. Representative approaches, such as personalized pangenome references [22, 23], aim to identify reference subgraphs or haplotype paths that better match the sequenced sample and thereby reduce mapping ambiguity caused by graph variants that are irrelevant to the sequenced sample. However, existing personalized reference methods mainly focus on individual samples and often rely on high-depth sequencing, accurate haplotype inference, or sample-specific graph construction [24]. Such requirements limit their applicability to large cohorts with limited sequencing depth, as constructing, indexing, and storing personalized references for each sample would substantially increase computational and storage costs. In addition, existing path selection strategies may overlook local source consistency in the pangenome graph [25]. Branch-node selection should not be treated simply as a set of independent local decisions, because adjacent branches can exhibit source-sharing relationships along the reference paths represented in the pangenome graph. If adjacent branch nodes are selected independently, the resulting path may contain abrupt switches between unrelated reference sources [26, 27]. Therefore, population-level reference recommendation requires not only an effective representation of the target population’s genetic background but also a mechanism to encourage local source consistency during branch-node selection.

In this study, we propose PGpath, a pangenome-based method for recommending population-adapted linear reference genomes. We aim to bridge the gap between pangenome graph representation and routine linear-reference analysis by generating a population-adapted linear reference from a selected pangenome subpath. First, we extract population-level k-mer frequency features from sequencing data to characterize the genetic background of the target population. We then reformulate reference recommendation as a branch-node prediction task in the pangenome graph, enabling the model to select candidate nodes that better match the sequence composition of the target population. Finally, we incorporate topology-aware constraints based on source-consistency relationships between adjacent branch nodes to improve local source-path consistency of the predicted branch selections. In this way, PGpath retains pangenome-derived sequence diversity while producing a linear reference compatible with standard downstream analysis pipelines. Experiments on population sequencing and variant detection benchmarks show that PGpath-derived references improve read mapping performance and reduce downstream variant detection errors, highlighting the potential of PGpath for large-scale population genomic analysis.

## Materials and methods

The workflow of PGpath consists of four main steps: (i) constructing a pangenome graph from high-quality and widely used reference assemblies and performing branch-node statistics (Fig. 1B); (ii) generating population-level sequencing meta-training data from diverse reference sources, followed by k-mer counting, filtering, and population-level feature construction (Fig. 1C); (iii) training a topology-constrained multi-task neural network to learn the relationship between population-level k-mer features and branch-node selection (Fig. 1D–F); and (iv) applying the trained model to target population sequencing data and integrating the predicted branch-node selections into a population-adapted linear reference sequence (Fig. 1G). PGpath can also be retrained using user-defined reference genome panels to support customized reference recommendation.

### Pangenome graph construction and branch-node statistics

We selected high-quality and widely used reference assemblies with diverse sequence backgrounds as the initial reference panel for pangenome graph construction. We denoted the initial reference set by $R=\left\{{R}_1,{R}_2,\dots, {R}_5\right\}$. The set comprised (i) the general human reference genome GRCh38 [28]; (ii) the telomere-to-telomere reference genome T2T-CHM13 [6]; (iii) the Genome in a Bottle (GIAB) high-confidence individual HG002 [29], derived from an Ashkenazi Jewish individual; (iv) the East Asian high-quality reference genome T2T-YAO [30], derived from a Han Chinese individual; and (v) the African ancestry representative reference NA19240 [31], derived from a Yoruba individual from the YRI population. The reference panel combines commonly used human references, benchmark individual assemblies, and high-quality assemblies from diverse genetic backgrounds, thereby providing broad sequence diversity for pangenome graph construction. We then constructed the pangenome graph from the above reference genomes using *Minigraph* [20], with T2T-CHM13 used as the backbone reference (Fig. 1B). The pangenome graph was represented as:


(1) \begin{eqnarray*} G=\left(S,L,\mathrm{P}\right) \end{eqnarray*}

where $S=\left\{{s}_1,{s}_2,\dots, {s}_n\right\}$ is the set of *n* sequence segments, *L* is the set of directed links between segments, and $P=\left\{{P}_1,{P}_2,\dots, {P}_m\right\}$ is the set of *m* reference paths. Each reference path is composed of an ordered series of sequence segments. If the out-degree of a segment ${s}_i\in S$ was greater than or equal to 2, the corresponding position was defined as a branch ${b}_i$. Specifically, branch ${b}_i$ consisted of all successor segments directly connected to ${s}_i$:


(2) \begin{eqnarray*} {\displaystyle \begin{array}{c}{b}_i=\left\{{s}_i^1,{s}_i^2,\dots, {s}_i^k\right\}\end{array}} \end{eqnarray*}

where *k≤ 5* denotes the number of candidate nodes at branch ${b}_i$. The successor segments of ${s}_i$ were defined as branch nodes of branch ${b}_i$. All branches in the graph were collected as:


(3) \begin{eqnarray*} {\displaystyle \begin{array}{c}B=\left\{{b}_1,{b}_2,\dots, {b}_l\right\}\end{array}} \end{eqnarray*}

The constructed pangenome graph contained 47 004 branches in total. Among them, 46 371 branches contained two branch nodes, 620 branches contained three branch nodes, and 13 branches contained four branch nodes.

### Generation of population-level sequencing meta-training data

To learn the relationship between population-level genetic features and branch-node selection in the pangenome graph, we constructed population-level sequencing meta-training data based on the reference genome set *R* (Fig. 1C). First, paired-end whole-genome sequencing data were simulated using ART [32], with each reference genome used as a template. The simulated sequencing depth was set to 3×, the read length was 75 bp, and the fragment length was 250 bp. For each of the five reference genomes, we generated 200 independently simulated sequencing samples.

To ensure independence between model development and evaluation, the simulated samples from each reference genome were partitioned into mutually exclusive training, validation, and test source pools at a ratio of 60%:20%:20% before population construction. Population-level units were then generated independently within each partition using the same mixed-source sampling strategy. Each simulated population consisted of 40 samples. One reference genome was designated as the major source, and its proportion was randomly sampled within the predefined 0.6–1.0 range. The corresponding number of constituent samples was drawn from this major source, while the remaining samples were drawn from the other reference sources within the same data partition. This design maintained a predominant population-level sequence background while preserving different degrees of within-population heterogeneity. In total, 600 training populations, 200 validation populations, and 200 test populations were generated. For each population, sample-level k-mer frequencies were averaged to obtain a population-level k-mer profile, and the most frequent branch node among the constituent samples was used as the target label at each branch. Feature selection and scaling were determined using the training data only, the validation set was used for model and hyperparameter selection, and the test set was reserved for final evaluation. Detailed procedures are provided in Supplementary S1.

Therefore, PGpath learns the mapping between population-level k-mer features and branch-node selection, enabling the extraction of a population-adapted linear reference path from the pangenome graph.

### Construction of the k-mer frequency matrix and feature selection

We used k-mer frequencies to characterize the sequence composition of population-level sequencing samples. In this study, *k* was set to 31, a commonly used setting in short-read genomic analysis that provides sufficient sequence specificity for distinguishing sequence differences across reference sources [33]. We evaluated the effects of different k-mer lengths on reference-source discrimination, with detailed results provided in Supplementary S2 We used *Jellyfish* [34] to count k-mers for each simulated population and constructed a global k-mer dictionary. To account for differences in sequencing data volume across populations, we normalized raw k-mer counts into k-mer frequencies (Fig. 2A). All data-dependent feature filtering and selection steps were performed using the training populations only. We first applied fixed sequence-based exclusion rules to remove k-mers containing ambiguous bases, repeat-associated k-mers, and k-mers derived from telomeric or centromeric repetitive regions. For the data-dependent low-frequency filter, the cumulative count of each remaining k-mer was calculated exclusively across the 600 training populations, and k-mers with training-population cumulative counts below 100 were removed (Fig. 2B). We then calculated between-group variance using only the training populations, ranked the remaining k-mers according to the variance of their mean frequencies across the five major-reference-source classes, and retained the top 5000 k-mers with the strongest between-group variability $Va{r}_m$:


(4) \begin{eqnarray*} {\displaystyle \begin{array}{c} Va{r}_m=\frac{1}{G-1}\sum_{g=1}^G{\left({\overline{x}}_{gm}-{\overline{x}}_m\right)}^2\end{array}} \end{eqnarray*}

where *G* denotes the number of population classes, ${\overline{x}}_{gm}$ denotes the mean frequency of the *m*-th k-mer in the *g*-th population class, and ${\overline{x}}_m$ denotes the overall mean frequency of the *m*-th k-mer across all populations. The candidate k-mers were then evaluated using one-way ANOVA, followed by Benjamini-Hochberg false discovery rate correction to control false positives from large-scale k-mer testing [35]. Finally, a total of 1967 population-discriminative k-mers with adjusted *q* values *≤ 0.01* were retained to construct the final feature matrix:


(5) \begin{eqnarray*} {\displaystyle \begin{array}{c}X\in{\mathbb{R}}^{N\times 1967}\end{array}} \end{eqnarray*}

where *N* denotes the number of population-level training samples (Supplementary S3). The feature matrix *X* was standardized and used as input to the subsequent topology-constrained multi-task neural network (Fig. 2C).

**Figure 2:**
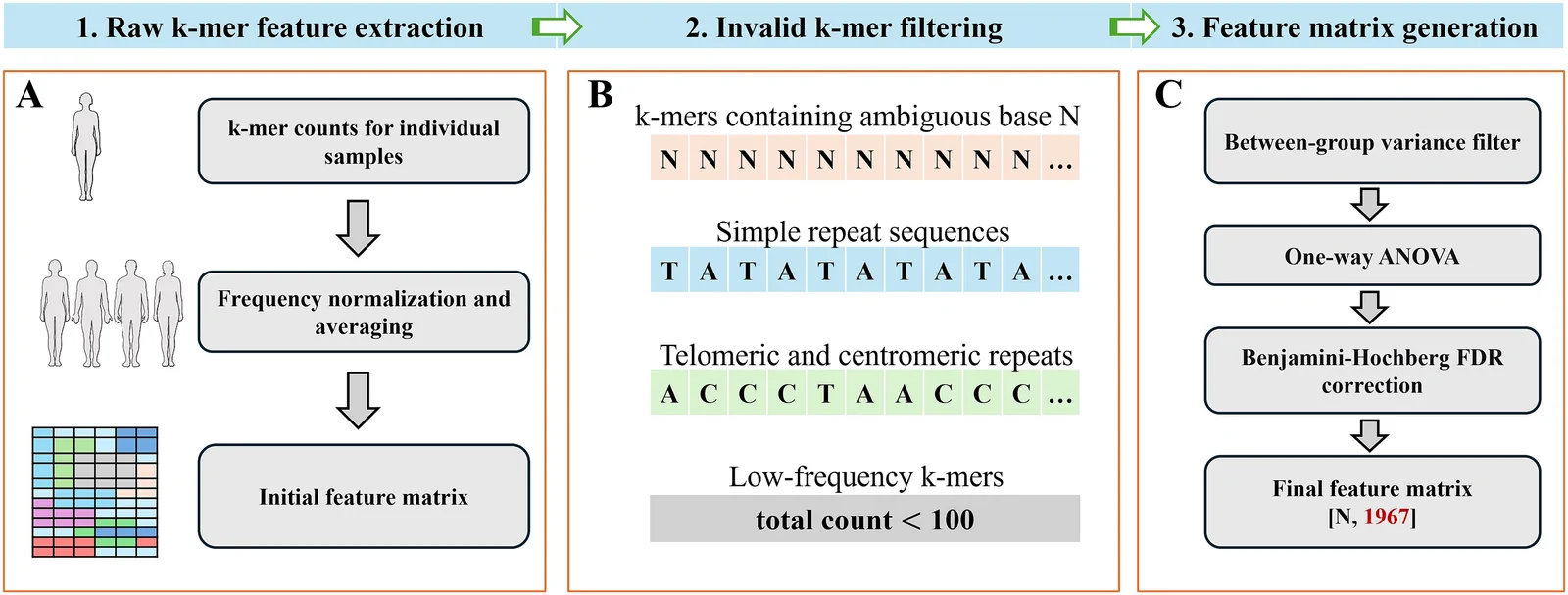
Construction of population-level k-mer feature matrix. (A) Raw k-mer feature extraction. Sample-level k-mer counts were normalized to frequencies and then averaged within each simulated population. (B) Filtering of invalid or uninformative k-mers. k-mers containing ambiguous bases, repeat-associated k-mers, k-mers from telomeric or centromeric repetitive regions, and low-frequency k-mers were removed before feature selection. (C) Generation of the final population-level feature matrix. Candidate k-mers were first screened by between-group variance across population classes, followed by one-way ANOVA and Benjamini-Hochberg FDR correction.

### Construction of branch-node topological associations

Branch-node selection in a pangenome graph should encourage local source consistency across adjacent branches. For a given sample or target population, if a branch node selected at one branch is derived from a specific reference source, the branch node selected at the next adjacent branch is more likely to come from the same reference source or from a node sharing that reference source. Therefore, source-consistency associations between adjacent branch nodes can be used as topological constraints to guide the model toward locally consistent branch-node selection.

We first identified adjacent branches according to their order along the pangenome paths. For two consecutive branches ${b}_i$ and ${b}_j$, if a branch node ${s}_i^u\in{b}_i$ and a branch node ${s}_j^v\in{b}_j$ shared at least one reference source, we defined a branch-node topological association between them. This association indicates that branch nodes ${s}_i^u$ and ${s}_j^v$are more likely to be jointly selected during population-adapted reference path recommendation. As shown in Fig. 1D, branch node ${s}_2$ is derived from ${ref}_{1,2,3,4}$, whereas branch node ${s}_4$ is derived from ${ref}_4$. Because the two nodes ${s}_2$and ${s}_4$ share the source ${ref}_4$, we connected them with an edge. Similarly, the source set of ${s}_7$ partially overlaps that of ${s}_2$, therefore, we also connected ${s}_2$ and ${s}_7$. Using this rule, we extracted 125 312 branch-node topological associations and constructed the branch-node association graph ${G}_{bn}$ (Fig. 1E). These associations encode a local source-sharing prior that is subsequently used to regularize branch-node predictions across adjacent branches.

### Topology-constrained multi-task neural network for branch-node prediction

We used a topology-constrained multi-task neural network to learn the relationship between population-level k-mer features and branch-node selection in the pangenome graph (Fig. 1F). The model takes two types of input: the standardized population-level k-mer feature matrix *X* and the branch-node association graph ${G}_{bn}$*.* Each branch-node selection in the pangenome graph is treated as a prediction task, and the model outputs a probability vector over the candidate branch nodes for each branch. The standardized k-mer feature matrix and the corresponding branch labels were split in the same manner to ensure sample-label consistency.

The training objective consisted of two components: a multi-task classification loss and a topological constraint loss. The multi-task classification loss supervised candidate branch-node selection for each branch and enabled the model to learn the correspondence between population-level k-mer features and branch-node labels. The loss was defined as:


(6) \begin{eqnarray*} {\displaystyle \begin{array}{c}{\mathcal{L}}_{ce}=-\sum_{b=1}^B{t}_b\cdotp \log{y}_b\end{array}} \end{eqnarray*}

where *B* denotes the number of branches, ${y}_b$ denotes the predicted probability vector for the *b*-th branch, and ${t}_b$ denotes the corresponding one-hot target label vector. To encourage local source consistency, we incorporated the branch-node association graph ${G}_{bn}$ into the training objective. Based on ${G}_{bn}$, we constructed the adjacency matrix ${A}_{bn}$, where an entry was set to 1 if two branch nodes were connected by a branch-node topological association and 0 otherwise. The corresponding graph Laplacian matrix was defined as:


(7) \begin{eqnarray*} {\displaystyle \begin{array}{c}{L}_{bn}={D}_{bn}-{A}_{bn}\end{array}} \end{eqnarray*}

where ${D}_{bn}$ denotes the degree matrix. The predicted probabilities of all candidate branch nodes were concatenated into a single probability vector *p*, and the topological smoothness loss was defined as:


(8) \begin{eqnarray*} {\displaystyle \begin{array}{c}{\mathcal{L}}_{smooth}={p}^T{L}_{bn}p=\sum_{\left(u,v\right)\in{E}_{bn}}{\left({p}_u-{p}_v\right)}^2 \end{array}} \end{eqnarray*}

where ${p}_u$ and ${p}_v$ denote the predicted probabilities at nodes *u* and *v*, respectively. The ${E}_{bn}$ is the edge set of the branch-node association graph ${G}_{bn}$. The ${\mathcal{L}}_{smooth}$ penalizes large prediction differences between topologically associated branch nodes, encouraging locally consistent branch-node selection across adjacent branches. The final loss function was defined as:


(9) \begin{eqnarray*} {\displaystyle \begin{array}{c}\mathcal{L}={\mathcal{L}}_{ce}+\mathrm{\lambda} \cdotp{\mathcal{L}}_{smooth} \end{array}} \end{eqnarray*}

where $\mathrm{\lambda}$ denotes the weight of the topological constraint. The model was trained for 500 epochs with a batch size of 64 and a dropout rate of 0.3. We used the Adam optimizer with an initial learning rate of $1\times{10}^{-3}$ and a weight decay of $1\times{10}^{-5}$. The validation set was used for hyperparameter selection, including the topology regularization weight, while the held-out test set was used only for final performance evaluation. Detailed training and reproducibility settings are provided in Supplementary S4.

For a target population, we extracted k-mer frequency features from sequencing data using the same procedure as in training, averaged sample-level k-mer frequencies into a population-level feature vector, and mapped it to the selected 1967-dimensional feature space. The standardized feature vector was then fed into the trained topology-constrained multi-task neural network to predict the candidate branch node for each branch in the pangenome graph. During reference reconstruction, we traversed the ordered backbone sequence and inserted a predicted non-backbone branch sequence only when it is connected to the current backbone region and can rejoin a downstream backbone node. Otherwise, the original backbone sequence is retained. This graph-connectivity check is applied during reference reconstruction independently of the topology regularization used during branch-node prediction. The resulting procedure generates a continuous population-adapted linear reference genome while incorporating population-relevant pangenome sequences. Detailed reconstruction procedures, including connector-node handling, anchor selection, fallback rules, independent graph-level reconstruction outcomes, and sensitivity analyses of the reconstruction limits, are provided in Supplementary S5 and Tables S1–S2, and the resulting reference can be directly used for downstream read mapping, variant detection, and population genetic analysis (Fig. 1G).

## Results

To evaluate the effectiveness of PGpath-derived population-adapted references, we first examined sequence differences between high-quality reference genomes from different genetic backgrounds, using the whole-genome comparison between T2T-YAO and T2T-CHM13 as an example. We then assessed whether the k-mers retained by the feature selection procedure captured reference-source-specific sequence composition, which provides the basis for representing population genetic background. Next, we evaluated the read mapping performance of PGpath-derived references on geographically diverse Human Pangenome Reference Consortium (HPRC) sequencing cohorts representing East Asian, European, and admixed American genetic backgrounds. We further investigated whether PGpath-derived references reduced downstream variant detection errors using the HG002 structural variant and small variant benchmark sets. Finally, we performed an ablation study to assess the contribution of the topology-aware constraint to branch-node prediction and local source-consistent transitions and analyzed the computational complexity, cohort-level amortization, and scalability of PGpath.

### Evaluation datasets and metrics

We used publicly available HPRC sequencing datasets for population-level read mapping evaluation and the GIAB HG002 benchmark for downstream variant detection evaluation. Three target sequencing cohorts were included in the read mapping analysis, representing East Asian, European, and admixed American populations. Each dataset contained five samples from the same continent or from populations with similar genetic backgrounds. The detailed sample composition is shown in Table 1.

**Table 1:** Sample information for the HPRC population datasets used in read mapping evaluation.

Dataset	Sample ID	Population	Description
**P-EAS** (East Asian)	HG00423	CHS	Han Chinese South
	HG00438	CHS	
	NA18538	CHB	Han Chinese in Beijing
	HG02364	CDX	Chinese Dai in Xishuangbanna
	HG02514	KHV	Kinh in Ho Chi Minh City
**P-EUR** (European)	HG00117	GBR	British in England and Scotland
	HG00118	GBR	
	HG00119	GBR	
	HG00272	FIN	Finnish in Finland
	HG00280	FIN	
**P-AMR** (Admixed American)	HG00733	PUR	Puerto Ricans from Puerto Rico
	HG00735	PUR	
	HG00738	PUR	
	HG01136	CLM	Colombians from Medellin, Colombia
	HG01138	CLM	

Read mapping was performed using *BWA-MEM*, and mapping statistics were summarized using *SAMtools*. Because different reference genomes have distinct coordinate systems, variants detected using different references were converted to a common coordinate framework before comparison with the GIAB benchmark. Specifically, *minimap2* and *wgatools* were used to establish coordinate correspondence between reference genomes, and *CrossMap* was then used for coordinate conversion. Read mapping quality was evaluated using four metrics: perfect match rate, gapless match rate, properly paired rate, and mapping quality (MAPQ) 60 rates. These metrics were determined from the NM tag, CIGAR string, SAM FLAG field, and MAPQ field, respectively, and their detailed definitions are provided in Supplementary S6. Downstream variant detection was evaluated against the GIAB HG002 high-confidence benchmark sets. After PASS filtering and the corresponding variant-class-specific processing, benchmark variants were restricted to the successfully harmonized common callable territories used consistently across all evaluated references. Structural variants were defined as variants with lengths of at least 50 bp, whereas small variants included SNPs and indels. The benchmark variant counts at the major filtering stages and the final truth sets used for evaluation are summarized in Table 2.

**Table 2:** GIAB HG002 benchmark variant counts at different stages of variant-detection benchmarking.

Benchmark set	Version	Initial variants^a^	PASS filtered^b^	Final benchmark truth set^c^
Structural variants (≥50 bp)	v0.6	74 012	12 745	8956
Small variants (SNPs & indels)	v4.2.1	4 048 342	4 022 526	3 794 534

^a^Initial variants refer to the raw variants downloaded from the GIAB repository.

^b^PASS filtered variants refers to variants retained after applying the PASS filter.

^c^Final benchmark truth set refers to the benchmark variants retained after the complete variant-class-specific processing, filtering, and restriction to the corresponding common callable territory described in Supplementary S11. The final small-variant truth set comprised 3 325 789 SNPs and 468 745 indels.

### Sequence differences between high-quality reference genomes

We first compared T2T-YAO with T2T-CHM13 at the whole-genome scale to assess reference differences associated with distinct genetic backgrounds. T2T-CHM13 provides a complete telomere-to-telomere reference assembly, whereas T2T-YAO represents a high-quality reference genome from an East Asian genetic background. Whole-genome alignment revealed clear chromosome-scale and local sequence differences between the two references. As shown in Fig. 3, the alignment of T2T-YAO against T2T-CHM13 exhibited heterogeneous coverage patterns across chromosomes. Many genomic regions displayed continuous collinear alignments, whereas local gaps, fragmented alignments, and discontinuous alignment patterns were observed in several regions, particularly around chr9, chr16, and telomeric or repetitive regions. Enlarged views further showed that several T2T-YAO segments lacked continuous alignments to the corresponding T2T-CHM13 coordinate system, suggesting sequence differences in structurally complex or repetitive regions. The alignment results indicate that high assembly quality does not eliminate sequence differences associated with distinct population backgrounds. A single linear reference genome is therefore insufficient to represent the full sequence diversity across populations, supporting the need for population-adapted reference genome recommendation.

**Figure 3:**
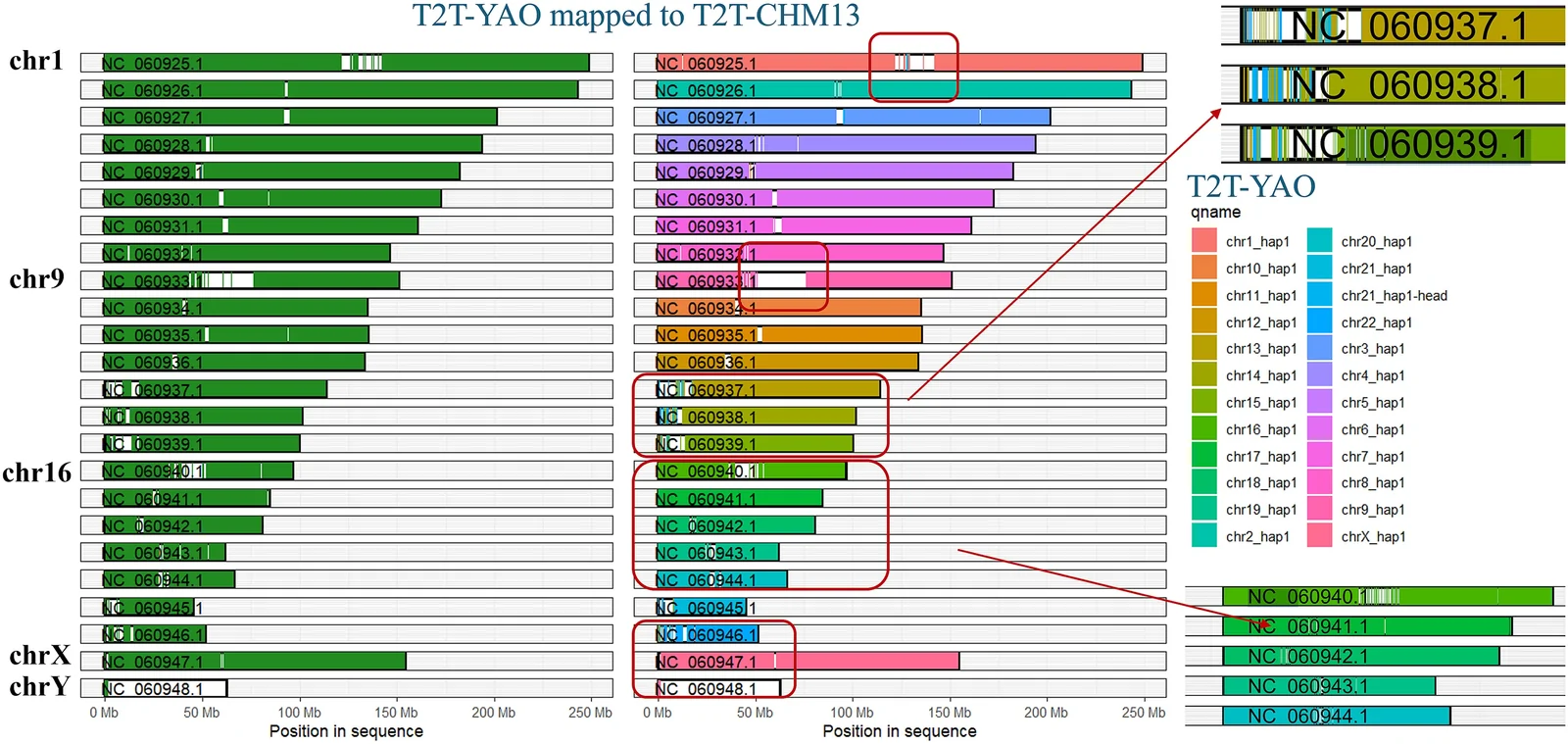
Whole-genome alignment between T2T-YAO and T2T-CHM13. T2T-YAO was aligned to T2T-CHM13 to evaluate sequence differences between two high-quality T2T-level reference genomes from different genetic backgrounds. Continuous colored blocks indicate genomic regions in which T2T-YAO sequences align continuously to the corresponding T2T-CHM13 coordinates, whereas blank gaps indicate large regions with no corresponding alignment or poor alignment. Red boxes and enlarged views highlight representative regions with evident alignment differences, including regions on chr1, chr9, chr16, chrX, and chrY.

We further performed pairwise alignments for all 10 possible combinations of the five human reference assemblies using minimap2 and evaluated their genome-wide collinearity and local alignment patterns, including alignment continuity, orientation, and block-length distributions. Although all comparisons showed extensive chromosome-scale collinearity, persistent local differences were observed in sequence content, alignment continuity, orientation, and the representation of complex regions, showing that assembly completeness alone is not a sufficient proxy for population representativeness and does not eliminate source-assembly-dependent sequence differences (Fig. S1; Supplementary S7).

### Population-discriminative k-mers can distinguish reference sources

We next evaluated whether the k-mers retained by the feature selection procedure could capture sequence composition differences across reference sources. The five most significantly differentiated k-mers were selected for visualization, and their frequency distributions were compared across samples derived from T2T-CHM13, GRCh38, HG002, T2T-YAO, and NA19240. As shown in Fig. 4, these k-mers exhibited clear reference-source-specific frequency patterns. For example, k-mer 1 showed a markedly higher frequency in GRCh38-derived samples than in samples from the other reference sources, whereas its frequency was relatively low in T2T-YAO-derived samples. k-mer 2 showed higher frequencies in NA19240- and T2T-YAO-derived samples but lower frequencies in T2T-CHM13- and HG002-derived samples. K-mer 3 and k-mer 4 also displayed distinct distribution patterns across reference sources. Overall, the selected k-mers showed relatively small within-source variation but pronounced between-source differences, indicating that the feature selection procedure retained k-mers that can distinguish reference sources and provide informative population-level signals for branch-node prediction.

**Figure 4:**
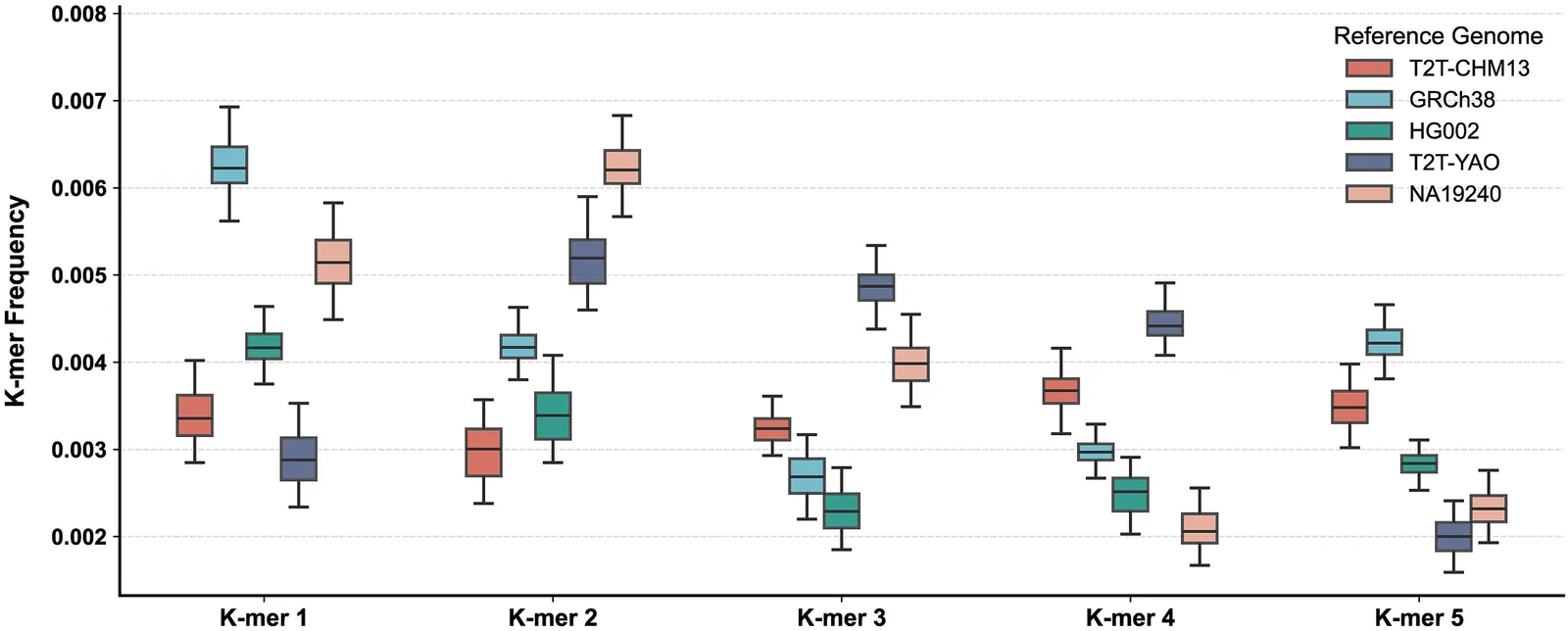
Frequency distributions of population-discriminative k-mers across reference genomes. Boxplots show the normalized frequency distributions of five representative population-discriminative k-mers across samples derived from T2T-CHM13, GRCh38, HG002, T2T-YAO, and NA19240.

We further assessed the robustness of the population-level k-mer representation to cohort size and reference-source admixture. Because each population profile is derived directly from the sequence content of the sampled genomes, cohort size primarily affects the stability of the aggregated population estimate. The results indicate that PGpath also remained robust across heterogeneous source compositions, with branch-node accuracy decreasing gradually as admixture increased and showing the largest reduction in highly balanced mixtures. Detailed analyses are provided in Supplementary S8 and Tables S3–S4. In addition, comparisons with simple reference-selection baselines confirmed that PGpath captured branch-level population-specific variation beyond dominant-source or nearest-reference selection, as detailed in Supplementary S9 and Tables S5–S11.

### PGpath-derived references can improve read mapping completeness

To evaluate whether PGpath-derived references improve read mapping performance on population sequencing data, we used three HPRC population datasets, including P-EAS, P-EUR, and P-AMR. For each target population, PGpath used the corresponding population-level k-mer frequency features to recommend a population-adapted linear reference from the full pangenome reference panel. The resulting population-adapted references were denoted collectively as PGpath-Ref(full) and the reads from P-EAS, P-EUR, and P-AMR were mapped to their corresponding PGpath-derived references, respectively. For comparison, reads from each dataset were also mapped to five fixed baseline reference genomes, including T2T-CHM13, GRCh38, HG002, T2T-YAO, and NA19240. Four read mapping metrics were evaluated: perfect match rate, gapless match rate, MAPQ 60 rate, and properly paired rate.

For perfect match and gapless match rates, PGpath-Ref(full) showed the highest mapping completeness across all three population datasets (Fig. 5A). In P-EAS, PGpath-Ref(full) achieved a perfect match rate of 78.064% and a gapless match rate of 98.372%, outperforming all baseline references. Similar patterns were observed in P-EUR and P-AMR, where PGpath-Ref(full) achieved the highest perfect match rates of 77.942% and 77.623%, respectively, and the highest gapless match rates of 98.441% and 98.228%, respectively. These results indicate that the population-adapted reference generated by PGpath can improve read-level sequence matching across geographically diverse populations. PGpath-Ref(full) also achieved the highest properly paired rate in all three datasets (Fig. 5C). The properly paired rates were 98.957% for P-EAS, 98.875% for P-EUR, and 98.412% for P-AMR, indicating improved paired-end mapping consistency in terms of read orientation and insert size.

**Figure 5:**
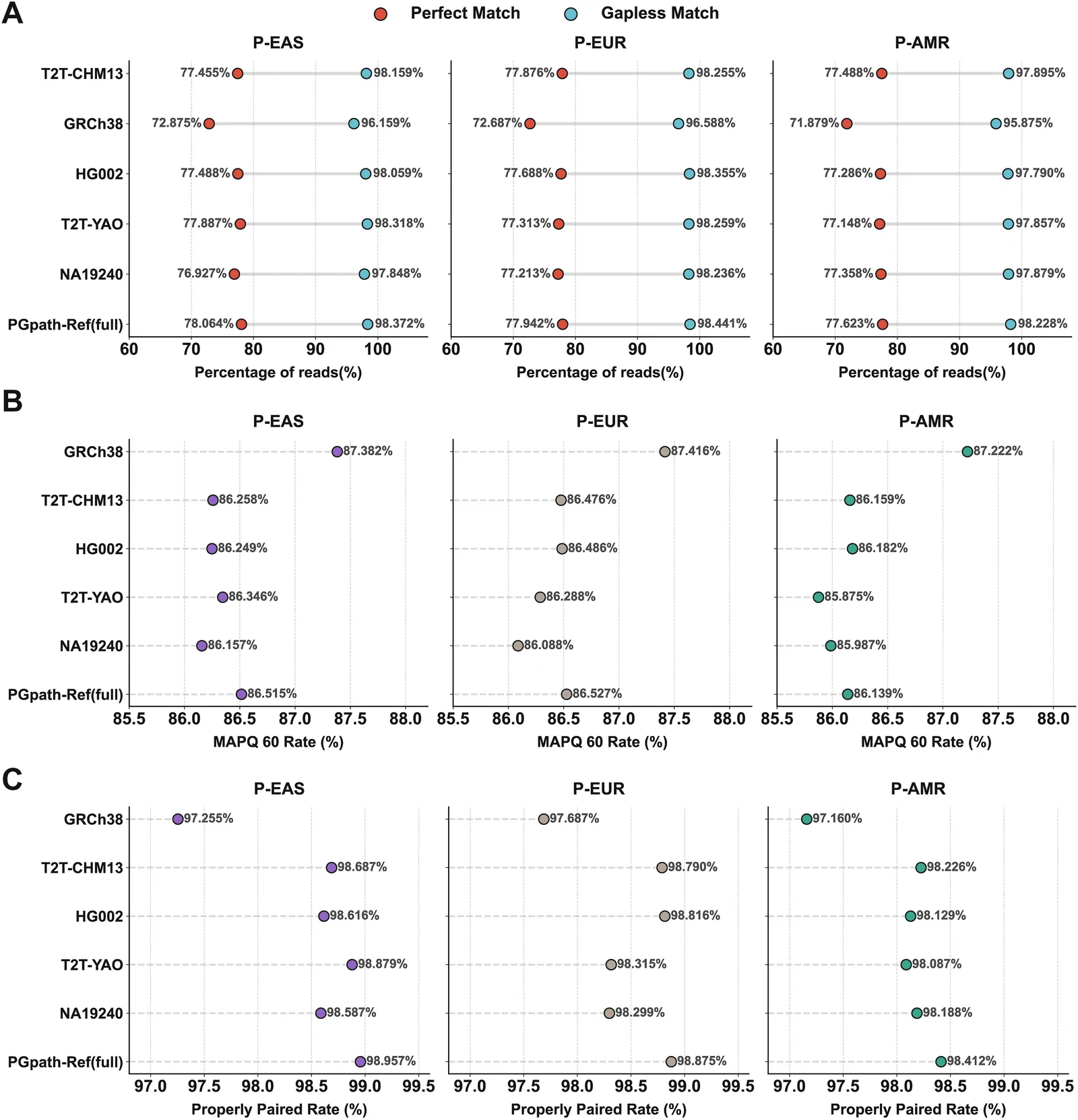
Read mapping performance of different reference genomes across population datasets. Read mapping performance was compared across three Human Pangenome Reference Consortium (HPRC) population datasets, including P-EAS, P-EUR, and P-AMR, using T2T-CHM13, GRCh38, HG002, T2T-YAO, NA19240, and PGpath-Ref(full) as reference genomes. (A) Perfect match rate and gapless match rate across different reference genomes. (B) Mapping Quality (MAPQ) 60 rate, defined as the proportion of reads with mapping quality score equal to 60. (C) Properly paired rate, reflecting the consistency of paired-end read mapping in terms of expected orientation and insert size.

The MAPQ 60 rate showed a different trend (Fig. 5B). GRCh38 achieved the highest MAPQ 60 rate across the three datasets, with values of 87.382%, 87.416%, and 87.222% in P-EAS, P-EUR, and P-AMR, respectively. This pattern may partly reflect differences in genome representation among the reference assemblies. One possible explanation is that unresolved or N-masked centromeric, telomeric, and repetitive regions in GRCh38 may cause reads originating from such regions to remain unmapped or to be excluded from the high-MAPQ category. Similarly, collapsed representations of highly similar multi-copy or segmental duplication regions could make some reads appear to map more uniquely, thereby increasing the apparent MAPQ 60 rate without necessarily indicating improved sequence representation completeness. Among the non-GRCh38 references, PGpath-Ref(full) consistently achieved a relatively high MAPQ 60 rate, reaching 86.514%, 86.527%, and 86.139% in the three datasets, respectively. Therefore, although GRCh38 showed the highest MAPQ 60 rate, PGpath-Ref(full) provided better read-level matching completeness and paired-end consistency across geographically diverse populations.

To evaluate whether the observed improvements were consistent across individuals rather than driven by a small number of samples, we further performed an individual-level paired analysis using the 15 HPRC individuals included in the evaluation. Instead of pooling reads within each population group, we calculated mapping metrics separately for each individual and compared the PGpath-derived reference with each fixed reference using paired differences. Across the three cohorts, PGpath-derived references showed consistent improvements across individuals, with positive paired differences observed for most evaluated metrics. Compared with GRCh38, improvements were observed across nearly all individuals, while comparisons with T2T-CHM13 showed smaller but still broadly distributed gains. The individual-level effect sizes, bootstrap confidence intervals, and the number of individuals showing improvement are reported in Supplementary S10 and Tables S12–S13.

### Sample-adapted leave-one-reference-out evaluation on HG002

We further performed a sample-adapted leave-one-reference-out (LOO) evaluation to examine whether PGpath could recommend an appropriate reference for an unseen individual and improve downstream variant calling. HG002 was selected because high-confidence GIAB benchmark sets are available for both structural variants and small variants. To avoid information leakage during model development, we completely removed HG002 from the reference panel and reran all stages of PGpath from scratch using only the remaining reference genomes, including pangenome graph construction, branch and topology definition, k-mer feature selection, feature standardization, model training, and hyperparameter determination. Thus, no information derived from the HG002 assembly was used during model development. After model construction, the HG002 sequencing profile was used as the inference input to generate a sample-adapted reference, denoted PGpath-Ref (LOO-HG002). The same HG002 sequencing data were then mapped to GRCh38, T2T-CHM13, T2T-YAO, NA19240, and PGpath-Ref (LOO-HG002) for downstream structural-variant and small-variant calling. This experiment represents a sample-adapted evaluation of generalization to an unseen genome and is distinct from the population-level evaluation described above.

For structural variants, we used Manta [36] and Delly [37]. Variant calls generated against different references were harmonized to the GIAB benchmark coordinate system and restricted to the same successfully transformed common callable territory before comparison. Unliftable or ambiguously mapped calls and unresolved coordinate inconsistencies were excluded during harmonization, and duplicate records were removed before benchmarking (Supplementary S11). Detailed call-level coordinate-harmonization statistics are provided in Table S14, and the conversion of GIAB benchmark regions and construction of the common callable territory are summarized in Table S15. The final evaluation used an identical truth denominator of 8956 SVs for all references, with truth and query denominators, TP, FP, FN, precision, recall, and F1 reported for each caller (Fig. 6A; Table S16). PGpath-Ref (LOO-HG002) achieved the highest F1 score with both callers: 94.18% with Manta and 94.53% with Delly, compared with 93.94% and 94.41%, respectively, for T2T-CHM13, the strongest fixed-reference baseline. GRCh38 showed substantially lower performance with both callers. These results indicate that PGpath-Ref (LOO-HG002) provides a favorable reference context for structural-variant detection across two independent SV callers.

**Figure 6:**
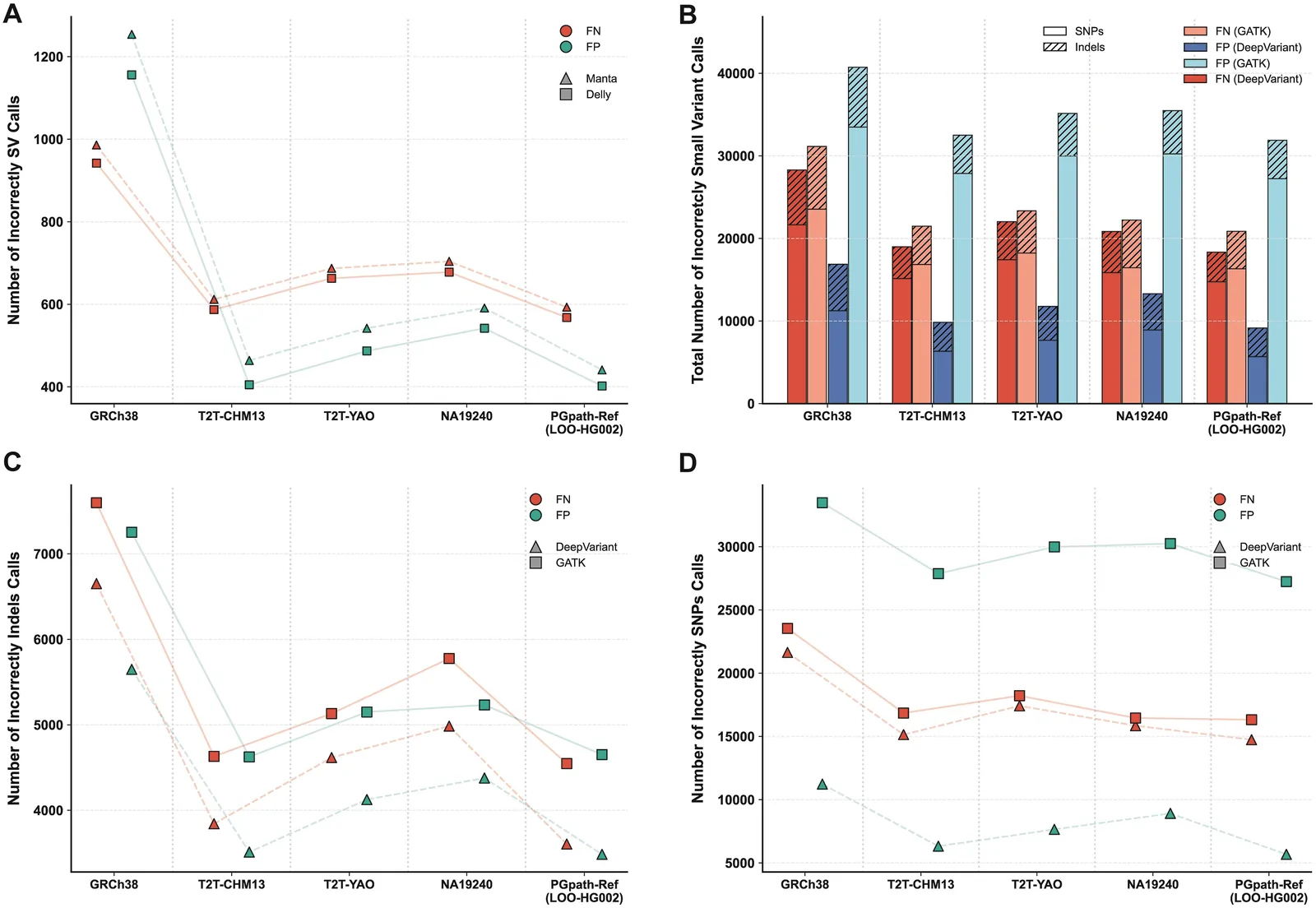
Variant detection errors using different reference genomes for HG002. Variant detection performance was evaluated using HG002 sequencing data mapped to GRCh38, T2T-CHM13, T2T-YAO, NA19240, and PGpath-Ref (LOO-HG002). Incorrect variant calls were quantified against the Genome in a Bottle (GIAB) HG002 high-confidence benchmark set. (A) False-negative and false-positive structural variant calls detected by Manta and Delly. (B) Total numbers of incorrectly detected small variants, including SNPs and indels, using *DeepVariant* and *GATK*. (C) False-negative and false-positive indel calls stratified by variant caller. (D) False-negative and false-positive SNP calls stratified by variant caller. FN, false negative; FP, false positive.

For small variants, we used DeepVariant [38] and GATK [39] and evaluated SNPs and indels separately. All callsets were represented in GRCh38 coordinates, normalized against the same reference FASTA, and restricted to the same common callable territory before benchmarking (Fig. 6B–D; Table S17). The resulting truth denominators were 3 325 789 SNPs and 468 745 indels for every evaluated reference. With DeepVariant, PGpath-Ref (LOO-HG002) achieved the highest F1 scores for both SNPs (99.69%) and indels (99.24%), compared with 99.68% and 99.22%, respectively, for T2T-CHM13. With GATK, PGpath-Ref (LOO-HG002) also achieved the highest SNP F1 score (99.35%), compared with 99.33% for T2T-CHM13. For GATK indels, the two references showed very similar performance: PGpath-Ref (LOO-HG002) achieved slightly higher recall (99.03% versus 99.01%) and F1 (99.02% versus 99.01%), while their precision values were essentially identical at the reported precision (99.01% for both). Overall, PGpath-Ref (LOO-HG002) showed consistently strong variant-detection performance across both small-variant callers and both variant classes within the same coordinate and callable-region framework.

### Ablation study of the topology-aware constraint

We further evaluated the contribution of the topology-aware constraint to branch-node prediction and adjacent source consistency. Based on the predicted branch-node labels of 200 held-out test samples, we calculated two metrics: mean test branch-node accuracy and mean adjacent source-consistency rate. Mean test branch-node accuracy measures the average proportion of correctly predicted branch nodes in each test sample. Mean adjacent source-consistency rate measures the average proportion of adjacent branch transitions in which the two predicted branch nodes share at least one reference source. Because the adjacent source-consistency rate directly reflects the source-sharing property encouraged by the topology regularizer, we interpret this metric as a diagnostic measure of whether the regularizer operates as intended. Adjacent branch pairs crossing chromosome boundaries were excluded from this calculation. When (*λ =0*), the topology-aware constraint was removed, and the model was trained only with the branch-node prediction loss. The ablation results are shown in Table 3.

**Table 3:** Effect of the topology-aware constraint weight in PGpath.

Model	$\mathrm{\lambda}$	Mean test branch-node accuracy (mean *±* SD%)	Mean adjacent source-consistency rate (mean *±* SD%^*^)
$\mathrm{PGpath}{\left(\mathrm{full}\right)}_{\mathrm{v}0}$	0	94.261 *±* 0.015	89.163 *±* 0.015
$\mathrm{PGpath}{\left(\mathrm{full}\right)}_{\mathrm{v}1}$	${10}^{-2}$	85.741 *±* 0.021	**95.793** *±* **0.017**
$\mathrm{PGpath}{\left(\mathrm{full}\right)}_{\mathrm{v}2}$	${10}^{-3}$	92.824 *±* 0.023	93.767 *±* 0.023
$\mathrm{PGpath}{\left(\mathrm{full}\right)}_{\mathrm{v}3}$	${10}^{-4}$	**95.958** *±* **0.017**	92.668 *±* 0.016
$\mathrm{PGpath}{\left(\mathrm{full}\right)}_{\mathrm{v}4}$	${10}^{-5}$	94.842 *±* 0.018	90.523 *±* 0.017

^*^SD, Standard Deviation.

Without the topology-aware constraint, PGpath achieved a mean test branch-node accuracy of 94.261% and a mean source-consistent transition rate of 89.163%. As the topology weight increased, the source-consistent transition rate generally improved, whereas excessively large weights reduced branch-node prediction accuracy. When $\lambda ={10}^{-2}$, the source-consistent transition rate reached the highest value of 95.793%, but the mean test branch-node accuracy decreased markedly to 85.741%, indicating that the topology constraint dominated the prediction objective and caused over-regularization, whereas a smaller weight of $\lambda ={10}^{-5}$ produced only limited improvement over the no-topology setting. In contrast, $\lambda ={10}^{-4}$ achieved the best overall balance, yielding the highest mean test branch-node accuracy of 95.958% while improving the source-consistent transition rate to 92.668%. Under this setting, the weighted topology loss was ~10%–15% of the cross-entropy loss during training, indicating that the topology-aware constraint provided an effective auxiliary regularization signal without dominating branch-node prediction. These results indicate that the topology-aware constraint improves local source-consistent transitions between adjacent branch predictions, and $\lambda ={10}^{-4}$ was selected as the default setting for PGpath.

We further evaluated the contribution of population-discriminative k-mer selection while fixing the topology regularization weight at $\lambda ={10}^{-4}$ (Table 4). Using all 5000-candidate k-mers without statistical selection reduced the mean test branch-node accuracy from 95.958% to 95.536%, despite providing substantially more input features. To distinguish the effect of feature selection from that of feature dimensionality, we additionally constructed a size-matched control using 1967 randomly selected candidate k-mers, which further reduced branch-node accuracy to 94.214%. A similar trend was observed for adjacent source consistency. These results indicate that the advantage of the selected k-mer representation cannot be explained simply by the number of input features.

**Table 4:** Ablation analysis of population-discriminative k-mer selection in PGpath.

k-mer setting	No. of k-mers	Mean test branch-node accuracy (mean *±* SD%)	Mean adjacent source-consistency rate (mean *±* SD%)
Between-group variance filter	5000	95.536 *±* 0.024	91.974 *±* 0.019
Random subset	1967	94.214 *±* 0.031	91.523 *±* 0.022
Selected k-mers (PGpath)	1967	95.958 *±* 0.017	92.668 *±* 0.016

Note: The topology regularization weight was fixed at the selected value of $\lambda ={10}^{-4}$ for all configurations. The random size-matched setting used 1967 k-mers randomly sampled from the 5000-candidate k-mers to control for feature dimensionality. All feature selection and preprocessing steps were performed using the training partition only.

We also analyzed the computational complexity, cohort-level amortization, and scalability of PGpath. The analysis indicated that PGpath performs pangenome-aware reference construction once per target population, enabling reference-construction and indexing costs to be amortized across the cohort while reducing redundant storage, maintaining coordinate consistency, and preserving compatibility with established linear-reference workflows. Further details are provided in Supplementary S12.

## Discussion

The selection of a reference genome directly affects the reliability of read mapping, variant detection, and downstream population genetic analysis. High-quality references such as T2T-CHM13 have greatly improved the completeness of human genome representation, but a single linear reference genome remains insufficient to capture genetic diversity across populations. Pangenome graphs provide a richer representation by integrating sequence variation from multiple reference sources. However, direct use of complete pangenome graphs in routine large-scale sequencing analysis is still limited by graph complexity, coordinate inconsistency, and insufficient compatibility with standard linear-reference workflows. PGpath was developed to translate pangenome diversity into a practical population-adapted linear reference by selecting a subpath from the pangenome graph, thereby retaining population-relevant sequence diversity while maintaining compatibility with conventional read mapping and variant detection pipelines.

A key feature of PGpath is that it reformulates reference recommendation as a branch-node prediction problem in the pangenome graph. Instead of constructing a sample-specific graph or directly using the full pangenome, PGpath learns the relationship between population-level k-mer frequency features and branch node selection. The selected k-mers showed clear reference-source-specific frequency patterns, indicating that population-level sequence composition can provide informative signals for reference recommendation. By using pooled population-level sequencing features rather than high-depth individual haplotype information, PGpath is better suited for large-scale cohort scenarios where per-sample reference construction would be computationally expensive. Another important design feature of PGpath is the incorporation of branch-node topological associations based on source consistency between adjacent branches. Branch-node selection should not be treated as a set of independent local decisions, as adjacent branches may belong to continuous segments or share reference-source consistency along graph paths. Independent branch-node prediction may introduce abrupt switches between unrelated reference sources and reduce the coherence of the generated reference path. PGpath uses source-consistency relationships between adjacent branch nodes as topological constraints in a multi-task neural network, encouraging locally consistent branch-node predictions. The topology-aware regularization promotes local source consistency between adjacent branch selections and reduces abrupt switches between unrelated reference-source paths.

Compared with existing personalized reference methods, PGpath emphasizes population-level applicability and ease of deployment. Personalized reference approaches often rely on high-depth sequencing, accurate haplotype inference, and sample-specific reference construction. These approaches are valuable for rare disease diagnosis, cancer genomics, and high-precision individual analysis, but they may face substantial cost and storage burdens in large-scale screening scenarios involving large cohorts and relatively low sequencing depth. At the methodological level, the two strategies also differ in their inference targets and outputs. Personalized approaches aim to obtain a reference that closely represents a particular individual and typically generate a separate reference or reference path for each individual. In contrast, PGpath seeks branch-node choices that represent the predominant sequence composition of the target population and reconstructs a shared population-adapted linear reference for that population. This population-level design allows reference construction and indexing to be performed once and the resulting reference to be reused across the entire cohort, thereby reducing redundant computation and storage while maintaining a common coordinate system. Therefore, PGpath provides a scalable intermediate strategy between a single universal reference and fully personalized references. Compared with a universal reference, PGpath improves population adaptability. Compared with per-sample personalized references, PGpath offers greater scalability and preserves compatibility with standard linear-coordinate analysis workflows.

The cohort-size and admixture sensitivity analyses further clarify the interpretation of the population-level representation used by PGpath. The model input is derived directly from the sequence content of the genomes sampled from a target cohort, and changing the number of sampled individuals does not alter the nature of this information. Instead, cohort size primarily affects how stably the underlying population-level sequence composition is estimated. Consistent with this interpretation, prediction stability increased as additional individuals were included, whereas the gain became progressively smaller once the cohort profile was represented by several individuals. The admixture analysis showed a similarly gradual response to increasing population heterogeneity rather than an abrupt loss of predictive ability. These observations suggest that PGpath does not depend on a fixed cohort size or a perfectly homogeneous population, although very small cohorts provide noisier estimates of the underlying sequence composition and highly balanced mixtures represent a more challenging setting.

The HG002 experiment addresses a distinct question from the population-level evaluations. In the simulated-cohort and HPRC analyses, PGpath uses information aggregated across multiple individuals to recommend a shared population-adapted reference. In contrast, the HG002 experiment uses the sequencing profile of a single individual after HG002 has been completely excluded as a reference source during model development. It should therefore be interpreted as a sample-adapted LOO evaluation of generalization to an unseen genome rather than as additional evidence for population-level performance. This distinction also illustrates that the PGpath framework can accept the same k-mer representation at different aggregation scales, while the biological interpretation of the resulting reference depends on whether the input profile represents an individual or a cohort.

Although PGpath showed promising performance in the current experiments, the number of reference genomes used for pangenome construction in this study remains limited. The current reference set includes several high-quality genomes, but it is still insufficient to cover the complex genetic diversity of global populations. It is important to emphasize that PGpath is a scalable tool. With the release of more high-quality T2T-level reference genomes and long-read assemblies, we can further expand the reference panel used by PGpath and improve coverage of diverse populations.

Overall, PGpath demonstrates the feasibility of automatically recommending population-adapted linear references from a pangenome graph. PGpath provides a reference genome construction strategy that balances population representativeness, linear-reference compatibility, and computational scalability for large-scale sequencing applications. It also provides a methodological foundation for future optimization of reference paths for target cohorts with shared genetic backgrounds, disease contexts, or other biologically relevant sequence characteristics.

**Key Points**

- PGpath is a scalable tool that converts population-relevant pangenome subpaths into population-adapted linear references compatible with standard read-mapping and variant-calling pipelines.
- Population-level k-mer profiles enable reference recommendation without high-depth haplotype inference or sample-specific construction, making the approach scalable for large sequencing cohorts.
- A topology-constrained multi-task network incorporates source-consistency relationships between adjacent branches as an auxiliary regularization signal for branch-node prediction.

## Supplementary Material

Supplementary_material_bbag547

## Data Availability

Instructions for use, source code, and analysis scripts for PGpath are available at https://github.com/Nevermore233/PGpath.
