# Supplementary material for: PGpath: recommending population-adapted linear reference genomes from pangenome subpaths
Source: Brief Bioinform. 2026 Oct 11;27(5):bbag547. doi: 10.1093/bib/bbag547 (PMC13755704; doi:10.1093/bib/bbag547)
Supplement: Supplementary file 1 [file supplementary_material_bbag547.zip › Figure S1.docx]

**Figure S1. Pairwise whole-genome alignment patterns among GRCh38, T2T-CHM13, HG002, T2T-YAO, and NA19240.** (A) Cumulative whole-genome dot plots for all ten pairwise comparisons. Forward-orientation alignments are black and reverse-orientation alignments are red. (B) Corresponding alignment block-length distributions on a log10 scale. Primary minimap2 alignments with block length >= 10 kb and MAPQ >= 20 were retained. The dominant diagonal in each comparison indicates broad chromosome-scale collinearity, whereas local gaps, fragmented or off-diagonal segments, and reverse-orientation alignments mark assembly- and region-dependent differences.

**Figure S1. Alt text:** Twenty-panel comparison of whole-genome alignments among GRCh38, T2T-CHM13, HG002, T2T-YAO, and NA19240. Panel A contains cumulative dot plots for all ten assembly pairs. Each plot has a dominant diagonal of forward-orientation alignments, indicating chromosome-scale collinearity, together with sparse off-diagonal and reverse-orientation segments marking local assembly-dependent differences. Panel B contains the corresponding alignment-length histograms on a logarithmic scale. The distributions are strongly right-skewed: most retained alignment blocks are between 10 kilobases and 100 kilobases, with progressively fewer blocks extending into the megabase range.
