# Supplementary material for: PGpath: recommending population-adapted linear reference genomes from pangenome subpaths
Source: Brief Bioinform. 2026 Oct 11;27(5):bbag547. doi: 10.1093/bib/bbag547 (PMC13755704; doi:10.1093/bib/bbag547)
Supplement: Supplementary file 1 [file supplementary_material_bbag547.zip › Supplementary-0916.docx]

**Supplementary Materials**

# **Supplementary 1. Source-level data partitioning and leakage-controlled model development**

To ensure independence between model development and evaluation, we partitioned the simulated sequencing samples at the source-simulation level before constructing any population-level units. This differs from partitioning the already constructed populations, because populations generated from a common source pool may contain the same underlying simulated samples even when the population units themselves are assigned to different data subsets.

For each of the five reference genomes, T2T-CHM13, GRCh38, HG002, T2T-YAO, and NA19240, we independently generated 200 paired-end whole-genome sequencing simulations using ART under the settings described in the main text. The 200 simulated samples from each reference source were randomly divided into three mutually exclusive subsets: 120 samples (60%) for training, 40 samples (20%) for validation, and 40 samples (20%) for testing. The split was performed independently for each reference genome and fixed before population construction. This resulted in 600 training-source simulations, 200 validation-source simulations, and 200 test-source simulations in total. No simulated sequencing sample was shared across the three source pools. Population-level units were subsequently generated separately within the training, validation, and test source pools. We constructed 600 training populations, 200 validation populations, and 200 test populations. The five reference genomes were equally represented as the major reference source, corresponding to 120, 40, and 40 populations per reference source in the training, validation, and test partitions, respectively. Each population consisted of 40 constituent sequencing samples. One reference genome was first designated as the major source, and a mixing coefficient was randomly sampled to determine the proportion of samples originating from that source. The remaining samples were drawn from the other four reference sources within the same data partition.

Within each population, constituent samples were drawn without replacement, so that the same simulated sample could not occur more than once in a single population. Because the objective of meta-training is to generate multiple populations with different source compositions, an individual source simulation could contribute to more than one population within the same partition. However, reuse was strictly restricted to that partition. Thus, a source simulation used to construct a training population could never contribute to a validation or test population, and the same constraint was applied to the other partitions. For each constituent sample, canonical 31-mer frequencies were calculated and normalized using the procedure described in the main text. The normalized frequency vectors of the 40 constituent samples were then averaged to obtain one population-level k-mer profile. Branch labels were generated independently for each population. At each pangenome branch, the frequencies of the candidate branch nodes among the 40 constituent samples were calculated, and the most frequent branch node was assigned as the target label.

All data-dependent k-mer feature-selection procedures were performed using the 600 training populations only. Specifically, construction of the candidate k-mer set, removal of invalid and low-frequency k-mers, ranking by between-group variance, selection of the top 5,000 candidate k-mers, one-way ANOVA, and Benjamini-Hochberg false discovery rate correction were performed without access to the validation or test populations. The resulting set of selected k-mers was then fixed and applied unchanged to the validation and test datasets. Feature standardization was also based exclusively on the training population matrix. For each selected k-mer, the feature-wise mean and standard deviation were estimated from the training populations and subsequently applied unchanged to the validation and test populations. Neither the feature set nor the scaling parameters were re-estimated using validation or test data. The topology-constrained multi-task neural network was trained using the training populations. The validation populations were used to select the topology-constraint weight and other model-selection settings. After the feature set, scaling parameters, model configuration, and hyperparameters had been fixed, the held-out test populations were used for final evaluation. The test data therefore did not contribute to source-sample construction for training, k-mer feature selection, feature scaling, model fitting, or hyperparameter selection.

In addition to this source-simulation-level separation, the HG002 leave-one-out analysis provided a more stringent genome-source-level evaluation. In this analysis, HG002 was excluded as an entire reference source before pangenome graph construction and model development. The pangenome graph, branch and topology definitions, simulated training data, k-mer feature selection, feature scaling, model training, and hyperparameter determination were reconstructed using only the remaining reference genomes.

# **Supplementary 2. Sensitivity of PGpath branch-node prediction to k-mer length**

The choice of k-mer length involves a trade-off among sequence specificity, robustness to sequencing errors, and computational complexity. Shorter k-mers generally occur more frequently across the human genome and may therefore provide less sequence-specific information, whereas longer k-mers increase sequence specificity but also enlarge the candidate k-mer search space and may be more sensitive to sequencing errors and local sequence variation. We used k = 31 as the default setting in PGpath because 31-mers are commonly used in short-read genomic analyses and provide a practical balance between sequence specificity and computational complexity [33]. This setting is also compatible with the 75-bp reads used in this study, for which each read contains multiple overlapping 31-mers.

We further evaluated the effect of k-mer length on PGpath branch-node prediction by testing k = 15, 21, 25, 31, and 35 using the same training, validation, and held-out test population partitions. For each k-mer length, candidate k-mers were generated independently, and all data-dependent feature-selection procedures were performed using the training partition only. The top 1,967 population-discriminative k-mers were retained for each setting so that the input feature dimensionality remained identical across all evaluated k-mer lengths. The resulting population-level k-mer profiles were then used as input to PGpath, while the model configuration and other training settings were kept unchanged across the different k values.

Performance was evaluated on the 200 held-out test populations across the 47,004 pangenome branches. For each test population, branch-node prediction accuracy was calculated as the proportion of the 47,004 branches for which the predicted node matched the corresponding target node. The resulting branch-node prediction accuracies were then summarized across the held-out test populations.

The mean branch-node prediction accuracies were 93.267% for k = 15, 93.423% for k = 21, 94.887% for k = 25, 95.958% for k = 31, and 95.674% for k = 35. The maximum difference across the tested k-mer lengths was 2.691 percentage points. Performance increased from k = 21 to k = 31, while k = 31 and k = 35 produced very similar results, differing by only 0.284 percentage points. Overall, PGpath branch-node prediction remained relatively stable across the evaluated k-mer lengths, particularly within the k = 25-35 range. Among the tested settings, k = 31 achieved the highest branch-node prediction accuracy, whereas increasing k to 35 provided little additional change in performance. We therefore retained k = 31 as the default setting because it provided strong branch-node prediction performance while maintaining a commonly used and computationally practical k-mer length for short-read genomic analysis.

# **Supplementary 3. One-way ANOVA and Benjamini-Hochberg false discovery rate control**

Following the source-level partitioning described in Supplementary 1, all k-mer feature screening and statistical testing were performed using the training populations only. The validation and test populations were not used for candidate k-mer ranking, statistical testing, or feature selection.

We statistically evaluated the candidate k-mer matrix produced after the between-group-variance filters. Let $X = \left( x_{im} \right)$ denote the $N \times M$ input matrix, where $N$ is the number of population-level samples, $M$ is the number of candidate k-mers entering the statistical test, and $x_{im}$ is the normalized frequency of candidate k-mer m in population-level sample $i$. Group membership was defined by the reference-source label encoded in each sample identifier, with each of the five classes containing 120 training population-level samples. Therefore, the statistical analysis in this section was based on 600 training populations in total. Before hypothesis testing, we standardized each candidate k-mer across the $N$ training population-level samples. For candidate k-mer $m$, the standardized value was calculated as:

$$\begin{aligned} z_{im}=\frac{\left( x_{im}- \mu_{m} \right)}{\sigma_{m}}, i = 1, \ldots, N; m = 1, \ldots, M\#\left( 1 \right) \end{aligned}$$

In Eq. (1), $\mu_{m}$ and $\sigma_{m}$ are the mean and standard deviation, respectively, of candidate k-mer $m$ across the training populations. This feature-wise z-score transformation placed all candidates on a common numerical scale. We then performed a separate one-way ANOVA for each candidate k-mer. Because the one-way ANOVA *F* statistic and its $P$ value are invariant to a nonzero affine rescaling applied separately to a feature, standardization did not alter the ANOVA ranking or $P$ values, apart from possible numerical-precision effects. Both $\mu_{m}$ and $\sigma_{m}$ in this statistical screening step were estimated exclusively from the training data.

For candidate k-mer $m$, the null hypothesis stated that its mean standardized frequency was equal across the G reference-source classes. The corresponding F statistic was calculated as the ratio of between-class variation to within-class variation:

$$\begin{aligned} F_{m}=\frac{\sum_{g=1}^{G} N_{g}\left( \bar{z}_{gm}-\bar{z}_{m} \right)^{2}/(G-1)}{\sum_{g=1}^{G} \sum_{i\in g} \left( \bar{z}_{im}-\bar{z}_{gm} \right)^{2}/(N-G)}\#\left( 2 \right) \end{aligned}$$

Here, $G$is the number of reference-source classes, $N_{g}$ is the number of population-level samples in class g, $\bar{z}_{gm}$ is the mean standardized frequency of k-mer m in class g, and $\bar{z}_{m}$ is its overall mean across all $N$ samples. In this study, $G = 5$, $N_{g} = 120$ in each class, and $N = 600$. Larger $F_{m}$ values indicate that the between-class difference in k-mer frequency is large relative to the variation observed among samples within the same class. We obtained one raw $P$ value, $p_{m}$, for each of the $M$ candidate k-mers from the F distribution with $G - 1$ and$N - G$ degrees of freedom.

Because M = 5,000 candidate k-mers were tested simultaneously, small $P$ values could arise by chance. We therefore adjusted the complete set of ANOVA *P* values using the Benjamini-Hochberg procedure. If $p_{1}\leq p_{2}\leq\ldots\leq p_{M}$ are the ordered $P$ values, the adjusted value for rank $i$ can be written as:

$$\begin{aligned} q_{\left( i \right)}=\min\left\{ \min_{j\geq i} \left( \frac{M}{j}p_{\left( j \right)} \right) \right\}\#\left( 3 \right) \end{aligned}$$

Adjusted values greater than 1 were set to 1. We retained a candidate k-mer when its Benjamini-Hochberg-adjusted q value was no greater than 0.01. Under the standard assumptions of the Benjamini-Hochberg procedure, the 1% threshold controls the expected proportion of false discoveries among the retained k-mers. It neither assigns each retained k-mer an individual 1% probability of being false nor controls the probability of making any false discovery across the complete experiment.

Of the 5,000 candidates, 1,967 k-mers satisfied $q_{m}\leq0.01$. Thus, the final feature count was determined by the observed data and the prespecified FDR threshold rather than by an arbitrary top-k cutoff. The original normalized-frequency columns, rather than the *z* scores used for statistical screening, were written to form the final feature matrix. The selected k-mer set was then fixed and applied unchanged to the validation and test populations. Model-input scaling parameters were also estimated from the training feature matrix only and subsequently applied unchanged to the validation and test data, as detailed in Supplementary 4.

# **Supplementary 4. Population-level k-mer profiling and branch-node inference**

We represent a target population with one population-level k-mer profile and use that profile to predict the candidate sequence node at each branch of the pangenome graph. Population profiling summarizes the aggregate sequence composition of the sampled individuals, whereas branch-node inference estimates which graph alternatives best match that composition. The reconstruction procedure described in Supplementary 5 subsequently tests whether the predicted alternatives form graph-valid substitutions within the backbone.

For each paired-end sequencing sample $s$, we count canonical 31-mers and retrieve the counts of the selected population-discriminative k-mers in the fixed order used during training. We normalize the selected counts within each sample so that samples with greater sequencing yield do not dominate the population profile. We define the frequency of k-mer $m$ in sample $s$ as:

$$\begin{aligned} f_{s,m}=\frac{c_{s,m}}{\sum_{l=1}^{K} c_{s,l}},m=1,\ldots,K\#\left( 4 \right) \end{aligned}$$

In Eq. (4), $c_{s,m}$ is the observed count of the *m*-th selected k-mer in sample $s$, $f_{s,m}$ is its normalized within-sample frequency, and $K$ is the number of selected k-mer features. For a target population containing $S$ sequencing samples, we calculate the arithmetic mean of the sample-level frequency vectors:

$$\begin{aligned} f_{\mathrm{pop},m}=\frac{1}{S}\sum_{s=1}^{S} f_{s,m}\#\left( 5 \right) \end{aligned}$$

Here, $S$ is the number of samples used to construct the target-population profile, $f_{\mathrm{pop},m}$ is the population-level frequency of k-mer $m$, and $s$ indexes the samples. Equal weighting ensures that each normalized sample contributes equally, rather than weighting samples according to their raw read counts. We then place the population vector in the training coordinate system by applying the feature-wise mean and scale learned from the training matrix:

$$\begin{aligned} x_{m}=\frac{f_{\mathrm{pop},m}-\mu_{m}}{\sigma_{m}}\#\left( 6 \right) \end{aligned}$$

In the standardization Eq. (6), $x_{m}$ is the standardized model input for the *m*-th feature, and $\mu_{m}$ and $\sigma_{m}$ are its mean and standard deviation estimated from the training data. We reuse the training values of $\mu_{m}$ and $\sigma_{m}$ without refitting them to the target population. Re-estimating the scaler from a single target-population vector would remove the relationship between the new profile and the training distribution.

We pass the standardized K-dimensional vector through two shared fully connected layers. The first layer maps K = 1,967 inputs to H = 1,024 hidden units, and the second layer maps H units to H/2 = 512 latent features. We apply batch normalization and ReLU activation after each transformation, and we use dropout only during training. A joint multi-task output layer then produces one probability distribution for each of the B = 47,004 branch tasks. For branch $b$ and candidate node $c$, we calculate:

$$\begin{aligned} p_{b,c}=\frac{\exp\left( z_{b,c} \right)}{\sum_{j\in C_{b}} exp(z_{b,j})}\#\left( 7 \right) \end{aligned}$$

In the *softmax* Eq. (7), $z_{b,c}$ is the model logit for candidate $c$ at branch $b$, $p_{b,c}$ is the corresponding probability, $C_{b}$ is the candidate-node set for branch $b$, and $j$ indexes the candidates in $C_{b}$. We select the candidate with the highest branch-specific probability and map the predicted class index to the corresponding GFA segment identifier defined in the training labels.

The model was trained for 500 epochs with a batch size of 64. The dropout rate in the shared feature extractor was set to 0.3. We used the Adam optimizer with an initial learning rate of $1\times{10}^{-3}$, a weight decay of $1\times{10}^{-5}$, and the default PyTorch settings for the remaining optimizer parameters ($\beta_{1}=0.9$, $\beta_{2}=0.999$, and $\epsilon={10}^{-8}$). The learning rate was adjusted using *ReduceLROnPlateau* with a reduction factor of 0.5 and a patience of 10 epochs. No early stopping was applied, and training was completed for the predefined 500 epochs. Model parameters were initialized using the default PyTorch initialization schemes. The random seed was set to 42 for Python, NumPy, and PyTorch to improve reproducibility. The 600 training populations were used for parameter optimization, the 200 validation populations were used for hyperparameter selection, and the 200 held-out test populations were reserved exclusively for final evaluation.

During inference, we use the trained parameters to predict all branch nodes in one forward pass. The resulting output contains GFA node identifiers rather than an assembled nucleotide sequence. We then pass the predicted node set to the backbone-guided reconstruction procedure, which verifies directed connectivity, downstream re-entry, chromosome consistency, and traversal limits before incorporating any non-backbone sequence.

# **Supplementary 5. Backbone-guided reconstruction of a continuous population-adapted reference**

PGpath converts the predicted branch nodes into a chromosome-resolved linear reference through a backbone-guided reconstruction procedure. The pangenome graph is parsed from the GFA file into segment sequences, directed graph links, and primary-backbone annotations. T2T-CHM13 is used as the primary backbone in this study, and primary nodes are ordered according to their chromosome and genomic offsets. Predicted non-backbone nodes are incorporated only when they can form a valid graph-supported alternative path that reconnects to the downstream backbone.

**5.1 Backbone-guided reconstruction procedure**

Reconstruction proceeds along each chromosome-specific backbone from left to right. At each backbone node, the current node is retained as the left anchor, and its outgoing graph edges are examined for predicted non-backbone nodes. If no predicted alternative is found, reconstruction proceeds to the next backbone node. If a predicted non-backbone node is available, alternative-path traversal is initiated. During alternative traversal, predicted successor nodes are preferentially followed when they are present among the outgoing graph edges. If no predicted successor is available, the traversal follows the next graph-supported outgoing node as a connector node. Connector nodes are intermediate graph nodes that are not directly selected by PGpath but are required to maintain a continuous directed path between the predicted alternative and the downstream backbone. This traversal strategy also allows nested branch structures to be followed when additional predicted branch nodes occur within an alternative path. When multiple outgoing candidates are available, the same deterministic graph order is used throughout reconstruction to avoid introducing additional optimization or path-search procedures.

The first primary-backbone node encountered during alternative traversal is treated as the candidate right anchor. An alternative path is accepted only when the candidate right anchor lies downstream of the left anchor on the same chromosome and the path satisfies the predefined reconstruction limits. Otherwise, the alternative is rejected and reconstruction continues along the original backbone. After an alternative is accepted, its sequence segments are incorporated and traversal resumes from the corresponding right anchor. This procedure avoids disconnected, backward, or unsupported inter-chromosomal paths while retaining the backbone sequence whenever a valid alternative path cannot be established. Two safeguards are applied during alternative-path traversal. The maximum skipped-backbone length is set to 500 kb, limiting the genomic interval that can be replaced by a single alternative path. The maximum alternative traversal is set to 1,000 graph nodes, limiting graph exploration in complex regions. These values are used as computational and graph-reconstruction safeguards rather than biological thresholds: the skipped-backbone limit reduces excessively long backbone replacements, whereas the traversal-step limit prevents unresolved paths from propagating through a large number of graph nodes. Alternatives exceeding either limit revert to the backbone path. The resulting reference remains linear and chromosome-resolved and can therefore be used directly with conventional read-mapping and variant-calling workflows.

**5.2 Independent graph-level reconstruction outcomes**

We further evaluated the graph-level reconstruction outcomes using the 200 held-out test populations. Predictions were obtained from two models trained under otherwise identical settings: the ablation model without the topology regularization term and PGpath with the default topology constraint. Predictions from both models were processed using the same backbone-guided reconstruction procedure and identical reconstruction parameters. For each population, an examined non-backbone alternative was defined as a predicted non-backbone branch encountered during backbone traversal and subjected to alternative-path reconstruction. An alternative was considered accepted when it could form a directed graph path, reconnect to an appropriate downstream backbone node on the same chromosome, and satisfy the skipped-backbone and traversal-step limits. Otherwise, it was classified as rejected/fallback, and reconstruction continued along the backbone. Thus, the number of rejected/fallback alternatives was calculated as the number examined minus the number accepted. Examined, accepted, and rejected/fallback counts were summarized across the 200 populations as mean ± standard deviation. The overall acceptance rate was calculated as the total number of accepted alternatives divided by the total number of examined alternatives across all 200 populations.

As shown in Table S1, the model without the topology constraint examined 3,173 ± 149 non-backbone alternatives per population, accepted 2,859 ± 158, and required fallback for 314 ± 25, corresponding to an overall acceptance rate of 90.104%. PGpath with the default topology constraint examined 3,264 ± 154 alternatives, accepted 2,987 ± 162, and required fallback for 277 ± 19, yielding an acceptance rate of 91.513%. Notably, the topology-constrained model examined slightly more non-backbone alternatives while producing fewer fallback events. The higher acceptance rate therefore was not associated with a reduction in alternative-path attempts; instead, a larger proportion of the examined alternatives satisfied the same downstream graph-reconstruction criteria. These results provide a complementary graph-level assessment of the predicted branch selections beyond the adjacent source-consistency metric.

The population-level target in PGpath is defined independently at individual branches according to sequence support among the constituent genomes, and individual graph nodes may be shared by multiple reference sources. Consequently, a unique reference-source path is not available for defining a conventional source-switch error, and sequence shared among multiple references cannot always be uniquely attributed to a single source without an additional attribution rule. The graph-level evaluation therefore focuses on directly observable reconstruction outcomes, including accepted and rejected alternatives, while the adjacent source-consistency measure is interpreted as a diagnostic of local source consistency rather than evidence of haplotype-level coherence.

**5.3 Sensitivity to reconstruction limits**

We further examined the sensitivity of backbone-guided reconstruction to the two predefined reconstruction limits using predictions from the final PGpath model on the same 200 held-out test populations. One parameter was varied at a time while the other was fixed at its default value. For the maximum skipped-backbone length, we evaluated 250, 500, and 1,000 kb while fixing the maximum traversal length at 1,000 nodes. For the alternative-traversal limit, we evaluated 500, 1,000, and 2,000 nodes while fixing the maximum skipped-backbone length at 500 kb. Reconstruction outcomes were evaluated using the same examined, accepted, rejected/fallback, and overall acceptance-rate definitions described above.

As shown in Table S2A, increasing the maximum skipped-backbone length from 250 to 500 and 1,000 kb increased the overall acceptance rate from 90.582% to 91.513% and 91.956%, respectively. Correspondingly, rejected/fallback alternatives decreased from 309 ± 21 at 250 kb to 277 ± 19 at 500 kb and 262 ± 20 at 1,000 kb. The additional increase from the default 500-kb setting to 1,000 kb was modest (0.443 percentage points), suggesting that the default setting accommodates most graph-supported local alternatives while retaining a conservative limit on long backbone replacements.

A similar pattern was observed for the traversal-step limit (Table S2B). The overall acceptance rates were 91.129%, 91.513%, and 91.784% at 500, 1,000, and 2,000 nodes, respectively, while the corresponding rejected/fallback counts decreased from 290 ± 20 to 277 ± 19 and 268 ± 19. Across the full 500-2,000-node range, the acceptance rate varied by 0.655 percentage points, indicating relatively limited sensitivity to this parameter within the evaluated range. The default 1,000-node limit therefore provides a practical intermediate setting that permits sufficiently long graph traversal while avoiding unnecessarily extended exploration of complex paths.

In summary, these analyses indicate that the backbone-guided reconstruction procedure incorporates predicted alternatives through explicit graph-connectivity and fallback criteria, and that reconstruction outcomes are relatively stable around the default 500-kb and 1,000-node settings.

# **Supplementary 6. Computational definitions of read-mapping metrics**

Read-mapping performance was quantified from the SAM/BAM files generated by BWA-MEM. All metrics were calculated at the read level. Secondary alignments (SAM FLAG 0x100) and supplementary alignments (SAM FLAG 0x800) were excluded so that each input read contributed at most one primary SAM record. For paired-end sequencing data, the two mates were counted separately as individual reads. Let $N_{total}$ denote the total number of input reads represented by primary SAM records, including both mapped and unmapped reads. The same denominator was used for all four read-mapping metrics.

Perfect match rate. A read was classified as a perfect match when its primary alignment was mapped and the edit-distance tag NM was equal to 0. The NM tag summarizes nucleotide differences between the read and the reference alignment, including mismatches and insertion/deletion events. Thus, NM = 0 indicates that no mismatch or indel was present in the aligned portion of the read.

Gapless match rate. Gapless matches were determined from the CIGAR string of each mapped primary alignment. A read was classified as gapless when its CIGAR string contained neither an insertion operation (I) nor a deletion operation (D); nucleotide mismatches were allowed.

Properly paired rate. Proper pairing was determined using the SAM FLAG field. For paired-end data, a read was classified as properly paired when FLAG 0x2 was set, indicating that the read and its mate were aligned as a proper pair according to the pairing criteria used by BWA-MEM, including the expected relative orientation and insert-size configuration.

MAPQ 60 rate. Mapping confidence was evaluated using the MAPQ field of the SAM record. A mapped primary read was classified as a MAPQ 60 read when its mapping quality score was exactly 60.

# **Supplementary 7. Pairwise whole-genome alignment patterns among five human reference assemblies**

To place the T2T-YAO versus T2T-CHM13 comparison in a broader reference context, we evaluated all ten pairwise combinations among GRCh38, T2T-CHM13 v2.0, HG002, T2T-YAO, and NA19240. We summarized each pair with a cumulative whole-genome dot plot and an alignment block-length distribution (Figure S1). Both panels were generated from the pairwise minimap2 PAF files, and the value of n shown in each histogram denotes the number of PAF alignment records included in the corresponding plotting dataset. Black segments represent forward-orientation alignments, red segments represent reverse-orientation alignments, and the axes represent cumulative positions in the query and target assemblies. Because the axes concatenate chromosomes, contigs, and scaffolds, the plots summarize genome-wide alignment structure but do not, by themselves, assign an off-diagonal segment to a specific rearrangement.

Across the ten comparisons, the cumulative plots showed a dominant diagonal extending through most of the genome length. The common diagonal indicates extensive chromosome-scale collinearity among the five assemblies despite differences in reference role, assembly strategy, and genetic background. Local gaps, changes in alignment density, short off-diagonal segments, and reverse-orientation segments interrupted the diagonal in every pair. The non-diagonal signals were concentrated in discrete intervals, consistent with greater representation differences in repetitive, centromeric, sub-telomeric, structurally complex, or assembly-specific regions.

The T2T-CHM13 versus T2T-YAO comparison contained 5,264 PAF alignment records in the plotting dataset (Figure S1). The cumulative plot preserved a strong chromosome-scale diagonal, while local interruptions and off-diagonal or reverse-orientation segments remained visible. The localized departures agree with the chromosome-specific gaps and fragmented patterns highlighted for chr9, chr16, and terminal or repetitive regions in main-text Figure 3. Assembly completeness therefore improves difficult-region coverage without making two references sequence-identical.

The four comparisons involving GRCh38 contained 6,630 alignments for GRCh38 versus T2T-CHM13, 6,989 for GRCh38 versus HG002, 5,027 for GRCh38 versus T2T-YAO, and 5,753 for GRCh38 versus NA19240. The six comparisons not involving GRCh38 contained 7,109 alignments for T2T-CHM13 versus HG002, 5,264 for T2T-CHM13 versus T2T-YAO, 5,105 for T2T-CHM13 versus NA19240, 5,258 for HG002 versus T2T-YAO, 5,327 for HG002 versus NA19240, and 4,843 for T2T-YAO versus NA19240. Thus, the numbers of PAF alignment records represented in Figure S1 ranged from 4,843 to 7,109 across the ten pairwise comparisons. The plots preserved a clear principal diagonal but also contained boundary-associated and off-diagonal signals. These patterns can reflect primary-sequence differences, alternate or unlocalized sequence representation, contig composition, and repetitive sequence organization. Forward-orientation alignments accounted for the majority in every comparison.

Alignment-length histograms were strongly right-skewed across all comparisons. Most PAF alignment blocks represented in the plots occurred between ${10}^{4}$ and ${10}^{5}$ bp, while progressively fewer alignments extended through the megabase and tens-of-megabases ranges. The shared distribution combines two signals: long blocks establish chromosome-scale correspondence, whereas numerous shorter blocks resolve local sequence similarity in fragmented or structurally complex regions. Alignment counts cannot be interpreted as direct genetic-distance measurements because contig fragmentation, duplicated sequence representation, and repeated local mappings also influence the number of blocks.

The pairwise analysis shows that the pattern observed for T2T-YAO and T2T-CHM13 is not unique to that pair. All five assemblies share a conserved genome-wide backbone but differ in local sequence content, alignment continuity, orientation, and complex-region representation. These findings indicate that assembly completeness alone is not a sufficient proxy for population representativeness.

# **Supplementary 8. Sensitivity of PGpath to cohort size and reference-source composition**

**8.1 Sensitivity to the number of individuals used to construct the population profile**

PGpath represents a target population using a population-level k-mer profile obtained by averaging the normalized k-mer frequencies across the sampled individuals. To evaluate the sensitivity of PGpath to cohort size, we kept the trained PGpath model, the selected k-mer features, and the training-derived standardization parameters unchanged, and varied only the number of individuals used to construct the population profile at the test stage. We evaluated representative cohort sizes of 1, 5, 10, 20, and 40 individuals. For each held-out 40-individual test population, smaller cohort profiles were generated by randomly subsampling the corresponding number of individuals without replacement and averaging their k-mer profiles. The resulting profiles were then provided to the same trained PGpath model without retraining or fine-tuning.

The population-level ground-truth label at each branch was defined from the complete 40-individual test population as the node receiving the greatest support among its constituent genomes. Branch-node prediction accuracy was calculated as the proportion of pangenome branches for which the predicted node matched the corresponding population-level ground-truth node. For cohort sizes smaller than 40, random subsampling was repeated to account for sampling variability, and the results are reported as mean $\pm$ SD in Table S3. Branch-node prediction accuracy increased progressively with cohort size, from 91.84 $\pm$ 0.03% using a single individual to 93.86 $\pm$ 0.03% with 5 individuals, 95.53 $\pm$ 0.02% with 10 individuals, 95.91 $\pm$ 0.02% with 20 individuals, and 95.96 $\pm$ 0.02% with 40 individuals. The largest improvement was observed when increasing the cohort from very small sample sizes, whereas the gain became progressively smaller as the cohort approached 40 individuals. These results indicate that incorporating additional individuals improves the representation of the population-level sequence signal, while the prediction performance becomes increasingly stable at larger cohort sizes.

**8.2 Sensitivity to reference-source composition**

We further evaluated the sensitivity of PGpath to variation in reference-source composition while fixing the population size at 40 individuals. For each fixed-composition setting, one of the five reference sources was selected as the designated major source and contributed 100%, 80%, 70%, 60%, 50%, or 40% of the individuals in the population. The remaining individuals were randomly sampled from the other four reference-source pools without imposing a fixed proportion among them. We additionally evaluated a random setting in which no major source was specified and the source of each individual was sampled with equal probability from all five reference-source pools. The trained PGpath model and all preprocessing parameters were kept unchanged throughout this analysis.

For every constructed population, the population-level ground-truth label at each branch was determined directly from its 40 constituent genomes: the node receiving the greatest actual support at that branch was used as the ground-truth node. We evaluated both branch-node prediction accuracy and the adjacent source-consistency rate, with results reported as mean $\pm$ SD in Table S4. As the major-source fraction decreased from 100% to 40%, branch-node prediction accuracy gradually decreased from 97.24 $\pm$ 0.02% to 93.73 $\pm$ 0.04%, while the adjacent source-consistency rate decreased from 93.82 $\pm$ 0.02% to 92.23 $\pm$ 0.03%. Under the random equal-probability sampling setting, branch-node prediction accuracy was 93.21 $\pm$ 0.06% and the adjacent source-consistency rate was 90.43 $\pm$ 0.03%. Thus, increasing heterogeneity in reference-source composition progressively increased the difficulty of branch prediction, but PGpath maintained a branch-node prediction accuracy above 93% across all evaluated settings. The accompanying decrease in adjacent source consistency indicates greater variability in local path composition in highly heterogeneous populations.

# **Supplementary 9. Comparison with simple reference-selection strategies and characterization of PGpath-derived references**

To determine whether the performance of PGpath could be explained by simple identification or reuse of one of the existing reference assemblies, we performed three progressively stronger baseline analyses. First, we selected the globally nearest reference according to population-level k-mer distance to test whether overall sequence similarity alone was sufficient for reference recommendation. Second, we used a simple multinomial logistic regression classifier to identify the major reference source and directly used its complete path, thereby testing whether PGpath primarily acts as a dominant-source classifier. Third, we constructed an independent per-branch majority baseline to evaluate whether simple branch-wise majority selection could reproduce PGpath without learning population-dependent branch relationships or incorporating topological constraints between adjacent branches. Finally, we characterized the reference-source support and sequence differences of the PGpath-derived references to quantify the extent to which they deviate from any single existing assembly. Together, these analyses distinguish global source identification, simple branch-wise selection, and the branch-specific, topology-aware reference adaptation performed by PGpath.

**9.1 Globally nearest reference selection based on population-level k-mer distance**

To examine whether the performance of PGpath could be reproduced by simply selecting the existing reference genome that is globally most similar to the target cohort, we constructed a nearest-reference baseline based on population-level k-mer distance. This baseline does not perform branch-node prediction or pangenome-path reconstruction. Instead, it identifies the reference source whose overall k-mer composition is closest to that of the target cohort and directly uses the corresponding complete reference genome for downstream analysis.

To avoid information leakage, reference-source k-mer profiles were constructed using the training source simulations only. For each of the five reference genomes, T2T-CHM13, GRCh38, HG002, T2T-YAO, and NA19240, 120 independently simulated sequencing samples were available in the training source pool. For each simulated sample, the normalized frequencies of the 1,967 k-mers retained by the training-only feature-selection procedure were extracted to form a *K*-dimensional feature vector, where K=1,967.

For reference source $s$, we averaged the k-mer frequency vectors of its $N$ training simulations to obtain a representative reference-source k-mer profile:

$$\begin{aligned} \mu^{(s)}=\frac{1}{N}\sum_{i=1}^{N} x_{i}^{(s)}\#\left( 16 \right) \end{aligned}$$

where ${x_{i}}^{(s)}$ denotes the K-dimensional k-mer frequency vector of the *i*-th training simulation generated from reference source $s$, and $\mu^{(s)}$ represents the mean k-mer profile of that reference source. This procedure generated five reference-source profiles, one for each candidate reference genome. For each target cohort $t$, including P-EAS, P-EUR, and P-AMR, sample-level normalized k-mer frequency vectors were calculated using the same procedure and averaged across the constituent sequencing samples to obtain one population-level k-mer profile, denoted by $x_{t}$. The target-cohort profile and the five reference-source profiles were then transformed using the feature-wise mean and standard deviation estimated from the training data only. Let $z^{(t)}$ denote the standardized profile of target cohort $t$, and $u_{z}^{(s)}$ denote the standardized profile of reference source $s$.

We quantified the global similarity between target cohort and reference source $s$ using Euclidean distance:

$$\begin{aligned} d_{t,s}=\left\| z^{(t)}-u_{z}^{(s)} \right\|_{2}=\sqrt{\sum_{m=1}^{K} \left( z_{m}^{(t)}-u_{z,m}^{(s)} \right)^{2}}\#\left( 17 \right) \end{aligned}$$

A smaller $d_{t,s}$ indicates greater overall similarity between the population-level k-mer composition of the target cohort and that of the corresponding reference source. The globally nearest reference for target cohort $t$ was therefore defined as:

$$\begin{aligned} s_{t}^{*}=arg\min_{s} d_{t,s}\#\left( 18 \right) \end{aligned}$$

where $s_{t}^{*}$ denotes the reference source with the smallest k-mer distance to target cohort $t$. Reference selection was based exclusively on the k-mer distance and was completed before downstream read-mapping results were examined.

The distances between each target cohort and all five candidate reference sources are reported in Table S5. The minimum distance in each row identifies the reference selected by the nearest-reference strategy. After the nearest reference was identified, its complete genome sequence was used directly as the reference; no local branch selection or reference reconstruction was performed. Because P-EAS, P-EUR, and P-AMR had already been mapped independently to all five candidate reference genomes using the same BWA-MEM pipeline, the read-mapping results corresponding to the selected nearest references were obtained from these existing mapping analyses. The nearest-reference strategy was then compared directly with PGpath-Ref(full) using perfect match rate, gapless match rate, MAPQ 60 rate, and properly paired rate. The comparison is summarized in Table S6.

PGpath-Ref(full) consistently achieved higher perfect-match and gapless-match rates than the globally nearest reference across the evaluated cohorts. These results show that the improvement of PGpath cannot be explained solely by identifying the existing assembly with the most similar overall k-mer composition and instead reflects additional branch-level reference adaptation.

**9.2 Major-source classification followed by complete reference-path selection**

To examine whether PGpath primarily identifies the dominant reference source of a population and subsequently reproduces the corresponding reference path, we constructed a second simple baseline based on major-source classification. In contrast to PGpath, this baseline does not perform branch-level prediction, multi-task learning, or topology-constrained path selection. Instead, it first predicts the major reference source of a target population from its population-level k-mer profile and then directly uses the complete path of the predicted reference source as the reference representation. Although this strategy preserves the internal continuity of an existing reference path, all branch decisions are constrained to a single globally selected source and therefore cannot capture local population-level deviations from that source. PGpath, in contrast, allows branch-specific adaptation while incorporating source-consistency relationships between adjacent branches to maintain local path coherence.

For each simulated population, the major reference source assigned during population construction was used as the class label. The five possible classes were T2T-CHM13, GRCh38, HG002, T2T-YAO, and NA19240. The input for each population was the same (K=1,967)-dimensional population-level k-mer feature vector used by PGpath. The k-mer feature set was selected using the training populations only, and all feature-standardization parameters were estimated from the training data and applied unchanged to the validation and test populations.

We used multinomial logistic regression as a simple supervised classifier. For a population with standardized k-mer feature vector $z$, the probability of assigning the population to reference source $s$ was calculated as:

$$P(y=s|z)=\frac{exp(w_{s}^{T}z+b_{s})}{\sum_{r=1}^{5} exp(w_{r}^{T}z+b_{r})}\#(8.4)$$

Where $w_{s}$ and $b_{s}$ are the coefficient vector and intercept for reference-source class *s*, respectively. The predicted major reference source was defined as the class with the highest predicted probability:

$$\begin{aligned} \hat{s}=arg\max_{s} P(y=s|z)\#\left( 8.5 \right) \end{aligned}$$

The classifier was fitted using the 600 training populations. The 200 validation populations were used to select the regularization strength, and the 200 held-out test populations were used only for final evaluation. Because the five reference sources were equally represented, the test set contained 40 populations for each major reference-source class.

We first evaluated whether the population-level k-mer features alone were sufficient to identify the designated major reference source. Classification accuracy was defined as the proportion of held-out test populations for which the predicted source $\hat{s}$ matched the designated major source $s$:

$$\begin{aligned} Source accuracy=\frac{\sum_{i=1}^{N_{test}} 1(\hat{s}_{i}=s_{i})}{N_{test}}\#\left( 8.6 \right) \end{aligned}$$

The classification results are summarized in Table S7.

We next examined whether accurate identification of the major reference source was sufficient to reproduce the branch-level reference decisions of the target population. For each held-out test population, once the major source $\hat{s}$ had been predicted, no further population-specific branch inference was performed. Instead, the complete pangenome path corresponding to the predicted reference source was used directly. The branch node traversed by this reference path at branch $b$, denoted by $c_{b}^{\hat{s}}$, was treated as the baseline prediction for that branch.

The target label $y_{b}$ at each branch was independently determined from the 40 constituent samples of the test population, as described in the main Methods, with the most frequent candidate branch node used as the population-level target. The branch-node accuracy of the major-source baseline for one test population was therefore calculated as:

$$\begin{aligned} BranchAccuracy=\frac{1}{B}\sum_{b=1}^{B} 1\left( c_{b}^{\hat{s}}=y_{b} \right)\#\left( 8.7 \right) \end{aligned}$$

where $B = 47004$ is the total number of branch prediction tasks, $c_{b}^{\hat{s}}$ is the branch node specified by the complete path of the predicted major reference source, and $y_{b}$ is the corresponding population-level target branch label. The final performance was summarized as the mean branch-node accuracy across the 200 held-out test populations. For comparison, PGpath was evaluated on exactly the same test populations and against the same branch labels. The results are summarized in Table S8.

The high classification accuracy demonstrates that the population-discriminative k-mer features contain sufficient information to identify the dominant reference source using a simple classifier. However, direct use of the complete path of the predicted major source produced lower branch-node accuracy than PGpath, showing that accurate source identification alone does not explain the branch-level predictions made by PGpath. The major-source baseline fixes all branch decisions to a single reference path, whereas PGpath captures local population-level deviations from that source through branch-specific prediction while incorporating source-consistency relationships between adjacent branches. At the same time, PGpath does not treat these local decisions as completely independent: source-consistency associations between adjacent branch nodes are incorporated as topological constraints to discourage abrupt switches between unrelated reference sources and preserve local path continuity. Such a result would therefore indicate that PGpath captures additional population-level variation at individual pangenome branches while maintaining coherence among adjacent branch selections, rather than simply identifying and reproducing the path of the dominant reference source.

**9.3 k-mer nearest-neighbor per-branch majority selection**

To further examine whether population-dependent branch-level adaptation could be achieved using a simple non-parametric strategy without the topology-constrained neural network, we constructed a k-mer nearest-neighbor per-branch majority baseline. Unlike the major-source complete-path strategy in Section 9.2, this baseline directly uses the k-mer composition of each target population and allows different branches to select different candidate nodes. However, each branch is predicted independently, without considering relationships between neighboring branches or applying any topology-based regularization.

The baseline was constructed using the 600 training populations only. All distances were calculated in the same standardized 1,967-dimensional k-mer feature space used by PGpath, with both the retained feature set and standardization parameters derived exclusively from the training data. For each validation or test population, we calculated its Euclidean distance to all 600 training populations and identified its k nearest training populations. The number of neighbors was determined using the validation set and fixed before evaluation on the held-out test set; k = 15 was used in the final analysis.

For each target population and each of the 47,004 pangenome branches, we independently examined the target branch labels of its 15 nearest training populations. The candidate node occurring most frequently among these neighboring populations was selected as the predicted node for that branch. When two or more candidate nodes had the same frequency, the tie was resolved according to a fixed node ordering. This procedure was repeated independently across all branches, allowing different branches to select nodes supported by different reference sources.

Importantly, this baseline does not use the designated major-source label of the target population. It relies only on similarity between the target population and the training populations in the standardized k-mer feature space. It also does not incorporate branch-node topological associations, topology loss, neighboring-branch information, or source-consistency preferences. Therefore, it provides a simple control for assessing whether population-specific branch selection can be reproduced by local k-mer similarity and independent branch-wise voting alone.

We evaluated the k-mer nearest-neighbor majority baseline and PGpath using branch-node accuracy and adjacent source-consistency rate (ASC). Branch-node accuracy was defined as the proportion of the 47,004 predicted branch nodes matching the corresponding population-level target branch labels. ASC was calculated as the proportion of adjacent predicted branch-node pairs that shared at least one supporting reference source in the pangenome graph, excluding transitions across chromosome boundaries. The results are summarized in Table S9.

The k-mer nearest-neighbor majority baseline achieved an overall branch-node accuracy of 94.83%, indicating that population-level k-mer similarity alone provides substantial information for branch-node selection. PGpath further increased the branch-node accuracy to 95.96%. A larger difference was observed in local path coherence: the nearest-neighbor majority baseline achieved an overall ASC of 90.63%, whereas PGpath reached 92.62%. Because the nearest-neighbor baseline performs an independent majority vote at each branch, it does not explicitly account for relationships between neighboring branch selections. In contrast, PGpath jointly learns population-dependent branch predictions from k-mer features while incorporating branch-node associations during model optimization. These results indicate that the performance of PGpath cannot be reproduced by a simple nearest-neighbor majority strategy based on population-level k-mer similarity alone.

**9.4 Reference-source support and sequence differences of PGpath-derived references**

To further characterize what PGpath contributes beyond selecting a single existing assembly, we examined the reference-source support and sequence differences of the PGpath-derived references reconstructed for P-EAS, P-EUR, and P-AMR. Specifically, we quantified the reference-source support of the branch nodes selected by PGpath and compared each reconstructed reference with the globally nearest existing reference identified in Section 9.1.

For each of the 47,004 pangenome branches, the node selected by PGpath may be supported by one or multiple reference paths. Because a candidate node can be shared by several reference genomes, assigning such a node exclusively to a single source would overstate its source specificity. We therefore used a fractional source-support scheme to summarize the reference support of each PGpath-derived reference. For a selected node supported by k reference sources, an equal contribution of 1/k was assigned to each supporting source. For example, a node shared by four reference paths contributed 0.25 to each of the four sources, whereas a node uniquely supported by one reference contributed 1.0 to that source. The fractional contributions were accumulated across all 47,004 selected branch nodes and normalized to obtain the reference-source support profile of each reconstructed reference. These values characterize the reference paths supporting the PGpath-selected nodes and should not be interpreted as estimates of population ancestry or admixture proportions.

We additionally classified each selected branch node as shared-support when it was traversed by two or more of the five reference paths and as single-source when it was supported by only one reference path. These two categories provide a complementary description of the multiplicity of reference support for the selected nodes. The resulting reference-source support profiles are summarized in Table S10. For P-EAS, the largest fractional contribution was associated with T2T-YAO (23.41%), followed by T2T-CHM13 (21.46%), HG002 (19.82%), GRCh38 (18.14%), and NA19240 (17.17%). For P-EUR, T2T-CHM13 showed the largest contribution (22.76%), followed by HG002 (21.43%), GRCh38 (20.21%), NA19240 (18.13%), and T2T-YAO (17.47%). For P-AMR, T2T-CHM13 also showed the largest contribution (22.32%), followed by HG002 (20.55%), NA19240 (19.69%), GRCh38 (18.78%), and T2T-YAO (18.66%). Thus, reference-source support was distributed across all five source genomes rather than being concentrated in a single reference path. The majority of PGpath-selected branches were supported by multiple reference paths. Shared-support branches accounted for 82.75% of P-EAS, 80.98% of P-EUR, and 80.56% of P-AMR, whereas single-source branches accounted for 17.25%, 19.02%, and 19.44%, respectively. This high proportion of shared-support branches reflects extensive sequence sharing among the five references represented in the pangenome graph. The single-source branches, in contrast, identify local regions in which the selected node is uniquely supported by one of the reference paths. Together, these results describe the support structure of the PGpath-selected nodes within the pangenome rather than assigning the reconstructed reference to a single source genome.

We next quantified the branch- and sequence-level differences between each PGpath-derived reference and its globally nearest existing reference identified by the population-level k-mer analysis in Section 9.1. At each of the 47,004 branches, the node selected by PGpath was compared with the node traversed by the corresponding nearest reference. A branch was classified as divergent when the two nodes differed and as matching when they were identical. We recorded the numbers and proportions of matching and divergent branches, the cumulative span of the PGpath-selected alternative sequences at divergent branches, and the net difference in reconstructed genome length relative to the nearest existing reference. The results are summarized in Table S11. For P-EAS, the globally nearest existing reference was T2T-YAO. Of the 47,004 pangenome branches, 42,205 were matching and 4,799 were divergent, corresponding to a divergent-branch proportion of 10.21%. These divergent branches involved approximately 14.21 Mb of PGpath-selected alternative sequence, and the reconstructed reference was 1.72 Mb longer than T2T-YAO. For P-EUR, the globally nearest reference was T2T-CHM13. PGpath retained 41,578 matching branches and selected alternatives at 5,426 branches (11.54%), involving approximately 16.83 Mb of alternative sequence and resulting in a net length increase of 2.18 Mb. For P-AMR, T2T-CHM13 was also the globally nearest reference. PGpath retained 41,321 matching branches and differed at 5,683 branches (12.09%), corresponding to approximately 18.56 Mb of alternative sequence and a net length increase of 2.45 Mb.

These results show that the PGpath-derived references retain substantial branch-level similarity to their globally nearest existing assemblies while introducing thousands of population-dependent alternative branch choices. Across the three target cohorts, 10.21-12.09% of the 47,004 branches differed from the corresponding globally nearest reference, involving 14.21-18.56 Mb of alternative sequence. The smaller net length differences of +1.72 to +2.45 Mb indicate that the branch substitutions include both length-increasing and length-reducing alternatives whose effects partially offset at the whole-reference level.

Together with the baseline analyses in Sections 9.1-9.3, these results clarify the contribution of PGpath beyond simple reference-source selection. Global k-mer similarity can identify an existing assembly that is broadly similar to the target population, while major-reference-source classification and k-mer nearest-neighbor voting capture substantial population-level sequence information. PGpath further performs population-dependent prediction at individual pangenome branches and incorporates relationships between branch-node selections during model optimization. Accordingly, the reconstructed references retain shared sequence where appropriate while introducing population-adapted alternative branch choices at specific regions of the pangenome, rather than simply reproducing the globally nearest existing assembly.

# **Supplementary 10. Individual-level mapping performance and uncertainty analysis across HPRC cohorts**

The read-mapping analysis in Figure 5 included 15 HPRC individuals from three population cohorts, with five individuals each from P-EAS, P-EUR, and P-AMR. To characterize the individual-level variability underlying the population-level summaries, we report the four-mapping metrics separately for each individual using T2T-CHM13, GRCh38, HG002, T2T-YAO, NA19240, and the corresponding PGpath-Ref(full). The evaluated metrics were perfect match rate, gapless match rate, MAPQ 60 rate, and properly paired rate. The complete individual-level values are provided in Table S12.

For perfect match rate, PGpath-Ref(full) produced higher values than GRCh38, HG002, T2T-YAO, and NA19240 for all 15 individuals and exceeded T2T-CHM13 for 14 of 15 individuals. For gapless match rate, PGpath-Ref(full) showed higher values than T2T-CHM13, GRCh38, HG002, and NA19240 for all 15 individuals and exceeded T2T-YAO for 14 of 15 individuals. For properly paired rate, PGpath-Ref(full) showed higher values in 13-15 of the 15 individuals, depending on the fixed reference. These individual-level results show that the higher perfect match, gapless match, and properly paired rates were broadly distributed across the evaluated samples rather than being driven by a small number of individuals. MAPQ 60 showed a different reference-dependent pattern. GRCh38 yielded higher MAPQ 60 rates than PGpath-Ref(full) for all 15 individuals, whereas PGpath-Ref(full) showed higher MAPQ 60 values than T2T-YAO and NA19240 for all 15 individuals and exceeded T2T-CHM13 and HG002 in 9 and 10 individuals, respectively.

To quantify the magnitude and uncertainty of these individual-level differences, we performed paired comparisons between PGpath-Ref(full) and each fixed reference within the same individual. For individual *i*, the paired difference was calculated as the mapping metric obtained with PGpath-Ref(full) minus that obtained with the corresponding fixed reference. The 15 paired differences were summarized using the mean difference in percentage points, a 95% bootstrap confidence interval, and the number of individuals with a higher PGpath value.

For perfect match rate, the mean paired differences between PGpath-Ref(full) and the five fixed references ranged from 0.270 to 5.396 percentage points, with all 95% bootstrap confidence intervals above zero. Higher PGpath values were observed in 14/15 to 15/15 individuals across the five comparisons. Gapless match rate showed mean paired differences of 0.202-2.140 percentage points, with higher PGpath values in 14/15 or 15/15 individuals. For properly paired rate, the mean paired differences ranged from 0.180 to 1.381 percentage points, with higher PGpath values in 13/15 to 15/15 individuals. For MAPQ 60, PGpath-Ref(full) showed positive mean paired differences relative to T2T-CHM13 (0.096 percentage points), HG002 (0.088), T2T-YAO (0.224), and NA19240 (0.317), whereas the mean paired difference relative to GRCh38 was -0.946 percentage points. The corresponding confidence intervals and individual-level directions are provided in Table S13.

In summary, Tables S12 and S13 provide the individual-level mapping values, paired effect sizes, and uncertainty estimates underlying the HPRC analysis in Figure 5. The paired results show that the higher perfect match, gapless match, and properly paired rates obtained with PGpath-Ref(full) were observed across most or all individuals in the three HPRC cohorts, while MAPQ 60 exhibited a distinct reference-dependent pattern.

# **Supplementary 11. Coordinate harmonization and common callable-territory benchmarking for HG002 variant detection**

This section provides detailed methodological and quality-control information for the HG002 variant-detection analysis presented in Figure 6. To ensure fair comparison of variant-calling performance across different reference genomes, we used a coordinate-harmonization and common-callable-territory framework. The evaluated references included GRCh38, T2T-CHM13, T2T-YAO, NA19240, and PGpath-Ref(LOO-HG002). SNPs and indels were called using DeepVariant and GATK, whereas structural variants (SVs) were called using Manta and Delly. Because these references use different coordinate systems and differ in the genomic regions that can be reliably transformed, variant calls and benchmark regions were harmonized to common coordinate systems and subsequently restricted to identical evaluable genomic territories before TP, FP, FN, precision, recall, and F1 were calculated. The detailed coordinate-conversion quality control, benchmark-region coverage, and denominator-based benchmarking results are provided below.

**11.1 Coordinate harmonization and variant-call quality control**

Separate GIAB benchmark sets were used for small variants and SVs according to their native coordinate systems. SNPs and indels were evaluated against the HG002 GRCh38 v4.2.1 benchmark and its corresponding high-confidence BED regions. Accordingly, DeepVariant and GATK callsets generated against alternative references were transformed to GRCh38 coordinates before comparison. SVs were evaluated against the HG002 Tier 1 v0.6 benchmark defined relative to GRCh37/hs37d5, and Manta and Delly callsets generated against the evaluated references were therefore represented in GRCh37/hs37d5 coordinates before benchmarking. The GIAB truth sets were retained in their native benchmark coordinate systems. Genome-to-genome coordinate mappings were generated from whole-genome alignments. Existing pairwise PAF alignments were reused when their orientation and base-level alignment information were appropriate, and additional alignments were generated using minimap2 when reciprocal mappings were required. PAF alignments were converted to UCSC chain format using wgatools and applied with CrossMap. Chain orientation and coordinate consistency were verified before variant and interval conversion. The coordinate-harmonization workflow used minimap2 2.1.1-r341, wgatools 1.1.0, CrossMap 0.7.3, bcftools 1.21, and bedtools 2.31.1.

For each caller, only variants passing the caller-specific PASS filter were entered into the coordinate-harmonization procedure. For SNPs and indels, records without a valid target coordinate were classified as unliftable, whereas records with more than one valid target location were classified as multiple mappings. Differences in REF alleles between assemblies were not automatically considered conversion failures because homologous loci can contain different reference alleles. After coordinate transformation, multiallelic variants were decomposed, left-aligned, and normalized against the corresponding benchmark reference FASTA using bcftools. Converted records whose normalized REF allele remained inconsistent with the target reference were classified as REF inconsistencies and excluded. Exact duplicate records generated during coordinate conversion and normalization were removed after successful harmonization and recorded separately from conversion failures.

SVs were processed separately because their representations can contain two breakpoints and coordinate-dependent fields such as END and SVLEN. For deletions, both breakpoints were required to map uniquely to the expected chromosome and form a valid interval in the target assembly. For insertions, the insertion breakpoint was transformed to the benchmark coordinate system. Coordinate-dependent fields were reconstructed after transformation. SVs with unmappable breakpoints, multiple target mappings, invalid breakpoint configurations, or unresolved coordinate inconsistencies were excluded, and exact duplicate records introduced during harmonization were subsequently removed.

The complete call-level conversion statistics are summarized in Table S14. For each callset, successfully harmonized records together with the mutually exclusive conversion-failure categories accounted for the PASS query variants before harmonization, whereas duplicates were recorded separately because they were removed after successful coordinate conversion. Small-variant harmonization success for the alternative references ranged from approximately 97.64% to 97.92%, while GRCh38 small-variant calls required no cross-assembly coordinate conversion. SV harmonization success across reference-caller combinations ranged from approximately 97.01% to 98.28%. These results indicate that most variant calls could be reliably represented in the corresponding GIAB benchmark coordinate systems, while ambiguous or unresolved transformations were explicitly excluded from subsequent benchmarking.

**11.2 Construction of common callable territories and benchmarking procedure**

Successful conversion of individual variant records does not by itself ensure a fair benchmark, because the amount of genomic territory that can be reliably represented may differ among references. We therefore independently constructed a common callable territory for small variants and SVs. For small variants, the analysis started from the HG002 GRCh38 high-confidence benchmark BED. Each benchmark interval was transformed from GRCh38 to an alternative reference and subsequently transformed back to GRCh38 using the reciprocal coordinate mapping. An interval was considered successfully represented only when it mapped uniquely and returned to the expected genomic location after reciprocal transformation. Regions that were unmappable, mapped ambiguously, were split into incompatible locations, or failed the reciprocal-coordinate check were excluded. The same procedure was applied to T2T-CHM13, T2T-YAO, NA19240, and PGpath-Ref (LOO-HG002), while GRCh38 served as the identity coordinate system. The SV callable territory was constructed using the same principle, starting from the GIAB Tier 1 v0.6 benchmark BED in GRCh37/hs37d5 coordinates. Because the SV benchmark is defined in hs37d5 coordinates, GRCh38 was treated as an alternative reference and subjected to the same reciprocal-conversion criteria as T2T-CHM13, T2T-YAO, NA19240, and PGpath-Ref(LOO-HG002). For each variant class, the final common callable territory was defined as the intersection of the benchmark regions that could be successfully and reciprocally represented across all five evaluated reference systems.

The reference-specific benchmark-region conversion results and the resulting common callable territories are summarized in Table S15. The original small-variant benchmark territory covered 2,516.87 Mb. After coordinate transformation and reciprocal validation, the intersection across all evaluated references yielded a common callable territory of 2,375.62 Mb, corresponding to 94.39% of the original benchmark territory. For SVs, the original benchmark territory covered 2,587.65 Mb, and the common callable territory covered 2,405.68 Mb, corresponding to 92.97% of the original SV benchmark territory. After the common callable territories were established, exactly the same callable BED was used for every reference and caller within the corresponding variant class. Both GIAB truth variants and harmonized query variants were restricted to these common regions before performance evaluation. SNPs and indels were evaluated using hap.py after all callsets had been represented and normalized in GRCh38 coordinates. SVs were evaluated using Truvari after transformation to GRCh37/hs37d5 coordinates. Identical SV matching criteria were applied to all Manta and Delly callsets, and the analysis was restricted to the insertion and deletion classes represented in the GIAB Tier 1 benchmark.

**11.3 Variant-detection performance within the common callable territories**

For each caller and variant class, Truth n denotes the number of GIAB benchmark variants within the common callable territory, whereas Query n denotes the number of harmonized query variants entering the same evaluation. Within the common SV callable territory, 8,956 GIAB benchmark SVs were available for evaluation, and this identical truth denominator was used for all evaluated references and both SV callers. The complete results are summarized in Table S16. With Manta, PGpath-Ref (LOO-HG002) achieved an F1 score of 94.18%, compared with 93.94% for T2T-CHM13, the strongest fixed-reference result. With Delly, PGpath-Ref(LOO-HG002) achieved an F1 score of 94.53%, compared with 94.41% for T2T-CHM13. Although the differences were modest, PGpath-Ref (LOO-HG002) maintained the highest F1 score with both SV callers when all references were evaluated using the same callable territory and truth denominator.

For small variants, restriction to the common callable territory resulted in identical truth denominators of 3,325,789 SNPs and 468,745 indels across all evaluated references. Detailed DeepVariant and GATK results are presented in Table S17. With DeepVariant, PGpath-Ref(LOO-HG002) achieved F1 scores of 99.69% for SNPs and 99.24% for indels, compared with 99.68% and 99.22%, respectively, for T2T-CHM13. With GATK, PGpath-Ref(LOO-HG002) achieved an SNP F1 score of 99.35%, compared with 99.33% for T2T-CHM13, and an indel F1 score of 99.02%, compared with 99.01% for T2T-CHM13. The differences among the strongest references were small, but PGpath-Ref (LOO-HG002) showed consistently competitive performance across both callers and variant classes.

In summary, Tables S14-S17 provide the detailed coordinate-conversion, quality-control, callable-territory, and denominator-resolved benchmarking information corresponding to the HG002 variant-detection analysis in Figure 6. Variant calls with unsuccessful or ambiguous coordinate transformations, unresolved reference-allele inconsistencies, or duplicate representations were explicitly handled before evaluation, and benchmark regions that could not be consistently represented across all evaluated references were excluded from the common callable territory. All reference-specific callsets were subsequently evaluated within the same genomic regions and against identical truth denominators. This framework ensures that differences in TP, FP, FN, precision, recall, and F1 reflect variant-detection performance within a common evaluation space rather than differences in the amount of genomic sequence available for benchmarking.

# **Supplementary 12. Computational complexity, cohort-level amortization, and scalability of PGpath**

The computational role of PGpath is broader than a small improvement in read-mapping or variant-calling accuracy. Its central systems-level property is that pangenome-aware reference selection is performed at the population level, after which one reconstructed linear reference and its indexes can be reused for all downstream samples assigned to that population. Compared with approaches that construct a separate reference for each individual, this population-level design amortizes reference construction and indexing costs across the cohort and avoids redundant per-individual reference storage. The following analysis characterizes asymptotic time, memory, and storage requirements and clarifies where PGpath sits between a universal linear reference, direct graph analysis, and fully personalized reference construction. The bounds describe algorithmic scaling rather than measured wall-clock superiority, and the actual performance depends on sequencing depth, graph density, hardware, storage throughput, and implementation details.

Let $N$ be the number of population-level training examples, $T$ the number of training epochs, K the number of selected k-mer features, $H$ the first hidden-layer width, $B$ the number of branch tasks, $C$ the maximum number of candidate classes per branch, and $R$ the number of nonzero entries in the sparse topology structure. In the current implementation, K = 1,967, H = 1,024, B = 47,004, and C ≤ 4. Ignoring constant factors introduced by the H/2 second hidden layer, one forward-backward training pass for one population example requires $O(KH + H^{2} + HBC)$ neural-network operations. Sparse multiplication by the graph Laplacian adds $O(R)$, yielding an overall offline training time of:

$$\begin{aligned} O\left[ TN\left( KH+H^{2}+HBC+R \right) \right]\#\left( 8 \right) \end{aligned}$$

The dominant model storage is $O(KH + H^{2} + HBC)$, while the padded branch output and sparse Laplacian require $O(BC + R)$ additional memory. Training is the largest learning-stage cost, but it is incurred only when the graph, feature set, or branch-label system is changed. It is not repeated for every target population and does not scale with the number of downstream samples analyzed after a model has been trained.

For a new target population, let $S$ be the number of sequencing samples used for profiling and $L$ the total number of sequenced bases in their FASTQ files. $K$ remains the number of selected k-mers. Read scanning requires work proportional to $L$, while querying, normalizing, and averaging the selected features requires work proportional to $SK$:

$$\begin{aligned} O\left( L+SK \right)\#\left( 9 \right) \end{aligned}$$

Eq. (9) implies that doubling the total amount of sequence data approximately doubles the counting work, assuming similar hardware and I/O conditions. We process samples sequentially and delete each temporary k-mer database after querying, so peak temporary disk usage is determined by the largest single-sample database rather than by the sum of all sample databases. After we construct the population profile, we run neural-network inference once for that population:

$$\begin{aligned} O\left( KH+H^{2}+HBC \right)\#\left( 10 \right) \end{aligned}$$

In the inference expression, $K$, $H$, $B$, and $C$ retain the definitions given above. The terms $KH$ and $H^{2}$ represent computation in the two shared layers, and $HBC$ represents computation in the branch-specific outputs. Let $Q$ denote the number of downstream samples assigned to the target population. $Q$ does not appear in EQ. (10) because we do not repeat branch-node inference for each downstream individual. We produce $B$ branch decisions once, and all $Q$ samples can subsequently use the same reconstructed reference.

We next parse and traverse the pangenome graph. Let $G$ be the total number of bases stored in graph segments, $V$ the number of segment records, $E$ the number of directed links, $M$ the number of ordered backbone nodes, $A$ the number of alternative-path nodes examined, and $F$ the number of bases written to the final FASTA. The ordinary reconstruction cost is approximately:

$$\begin{aligned} O\left( G+V+E+M\log M+A+F \right)\#\left( 11 \right) \end{aligned}$$

The reconstruction expression is near-linear in the graph and output sizes. In practical terms, a larger graph or a longer output sequence requires proportionally more reading, traversal, and writing. If many invalid alternatives are repeatedly tested, interval-length checks can add extra work, but the imposed traversal and skipped-backbone limits keep every individual search bounded.

The cohort-level benefit becomes clear after we combine the population-adapted stages. Let $C_{\mathrm{profile}}$, $C_{\mathrm{infer}}$, and $C_{\mathrm{reconstruct}}$ denote the costs of population profiling, inference, and reconstruction, and let $C_{\mathrm{build}}$ be their sum. In the following equations, $C(\cdot)$ denotes a computational cost and is unrelated to the candidate-class count C used above:

$$\begin{aligned} C_{\mathrm{build}}=C_{\mathrm{profile}}+C_{\mathrm{infer}}+C_{\mathrm{reconstruct}}\#\left( 12 \right) \end{aligned}$$

If $C_{\mathrm{linear}}$ denotes the cost of analyzing one sample with a conventional linear-reference workflow and $Q$ is the number of downstream samples in the cohort, total and average costs are:

$$\begin{aligned} C_{\mathrm{total}}\left( \text{PGpath},Q \right)=C_{\mathrm{build}}+QC_{\mathrm{linear}}\#\left( 13 \right) \end{aligned}$$

$$\begin{aligned} C_{\mathrm{avg}}\left( \text{PGpath},Q \right)=C_{\mathrm{linear}}+\frac{C_{\mathrm{build}}}{Q}\#\left( 14 \right) \end{aligned}$$

In these two equations, $C_{\mathrm{total}}$ is the total cost for $Q$ samples and $C_{\mathrm{avg}}$ is the average cost per sample. The term $C_{\mathrm{build}}/Q$ is the reference-building overhead assigned to one sample. Increasing Q reduces that overhead because the same population reference is reused. A personalized-reference strategy usually incurs a separate construction and indexing cost for every individual, so its per-sample reference-building overhead does not decrease through cohort reuse.

Reference storage follows the same principle. Let $P$ be the number of target populations and $S_{\mathrm{ref}}$ the storage required for one linear reference and its indexes. We store one reconstructed reference per target population, so our storage requirement scales with $P$, whereas per-individual reference storage scales with $Q$:

$$\begin{aligned} S_{\mathrm{population}}=O\left( PS_{\mathrm{ref}} \right);S_{\mathrm{individual}}=O\left( QS_{\mathrm{ref}} \right)\#\left( 15 \right) \end{aligned}$$

In the storage expression, $S_{\mathrm{population}}$ is the total storage for population-level references and their indexes, whereas $S_{\mathrm{individual}}$ is the corresponding total for per-individual references. Typical cohort studies have far fewer target populations than individuals, so $P$ is usually much smaller than $Q$. Population-level reuse also provides a shared coordinate system for joint calling, annotation, quality control, and cross-sample comparison. The shared-coordinate benefit reduces operational complexity even when wall-clock runtime is not the only concern.

The theoretical bounds describe how resource use grows with data, model, graph, and cohort size. To complement this theoretical analysis, we further evaluated the empirical runtime and memory consumption of the major PGpath computational stages under fixed hardware configurations. GPU-based model training and inference were performed on a server equipped with an AMD EPYC 7443P 24-Core Processor, 256 GB system memory, and eight NVIDIA Tesla V100 16-GB SXM2 GPUs. Each PGpath training or inference run used a single V100 GPU. CPU-based k-mer profiling and reference reconstruction were performed on a server equipped with an Intel Xeon Platinum 9242 CPU at 2.30 GHz, 96 CPU cores, and 384 GB system memory. All runtime and memory measurements reported below were obtained under these specified hardware environments. Because wall-clock time and memory consumption can vary with hardware configuration, software versions, storage performance, thread configuration, and concurrent system load, these values should be interpreted as empirical computational costs under the tested configuration rather than hardware-independent performance bounds.

(1) Model training. Using 600 training populations, 500 epochs, and a batch size of 64, PGpath required approximately 44-47 min to complete one training run. Peak host-memory usage was approximately 6.5 GB, and peak GPU memory allocation was approximately 2.8 GB. Model training represents an offline, one-time computational cost for a fixed pangenome graph, selected feature set, and branch-label system, and therefore does not need to be repeated for each target population. (2) Population k-mer profiling. For one approximately 30× whole-genome sequencing sample, processing from FASTQ input through Jellyfish k-mer counting, selected-k-mer extraction, feature normalization, and subsequent population-profile preparation required approximately 18-21 min per sample. Peak host-memory usage was approximately 14.0 GB. Population-level averaging of the selected k-mer profiles required only a small additional computational cost. Because individual sequencing samples can be processed independently, this stage can be parallelized across samples when sufficient CPU resources are available. (3) Population-level branch-node inference. Once the population-level k-mer profile had been prepared, end-to-end inference for one target population required approximately 21-24 s. This measurement included loading the required model resources, feature standardization, neural-network inference, decoding predictions for all 47,004 branches, and writing the prediction output. Peak host-memory usage was approximately 3.2 GB, and peak GPU memory allocation was approximately 0.6 GB. Branch-node inference is performed once for each target population and is not repeated for every downstream individual assigned to that population. (4) Reference reconstruction. Reconstruction of a chromosome-resolved linear reference from the complete branch-node prediction set and the pangenome GFA required approximately 30 s for one target population, with a peak host-memory usage of approximately 18.0 GB. This stage was performed entirely on the CPU and did not require GPU resources. Once reconstructed, the resulting population-adapted linear reference can be indexed once and reused across all downstream samples assigned to that population. Thus, after preparation of the population-level k-mer profile, the PGpath-specific inference and reconstruction procedures together required approximately one minute under the tested hardware configuration.

Pangenome graph construction was not included in these runtime measurements because it is an upstream, one-time preprocessing step performed using Minigraph and is not repeatedly executed when PGpath is applied to new target populations. Once constructed, the same pangenome graph can be reused for model training, population-level inference, and reference reconstruction.

Taken together, the theoretical analysis and empirical measurements characterize the computational behavior of PGpath from complementary perspectives. The theoretical bounds describe how computational requirements scale with sequencing data, model size, graph structure, and cohort size, whereas the empirical measurements provide practical runtime and memory estimates under defined hardware configurations. These results support the cohort-level amortization of reference construction, reference and index reuse, coordinate consistency, and compatibility with established linear-analysis tools. We do not claim absolute speed superiority over a specific graph aligner or personalized-reference method without a controlled empirical comparison. Our workflow occupies an intermediate computational position: we retain pangenome-aware sequence selection during reference construction and then return routine cohort analysis to a reusable linear-reference workflow.
